# High-Altitude Residence, Blood Biomarkers, and Chronic Disease Risk in Middle-Aged and Older Chinese Adults

**DOI:** 10.3390/healthcare14162554

**Published:** 2026-08-15

**Authors:** Han Shi, Yizhan Wu, Jiangwei Liu

**Affiliations:** 1 Department of Graduate School, Xinjiang Medical University, Urumqi 830000, China; shihan@stu.xjmu.edu.cn (H.S.); wuyizhan@stu.xjmu.edu.cn (Y.W.); 2 Key Laboratory of Special Environmental Medicine of Xinjiang, General Hospital of Xinjiang Military Command, Urumqi 830000, China

**Keywords:** residential elevation, chronic disease, blood biomarkers, restricted cubic spline, mediation analysis

## Abstract

**Background/Objectives**: The goal of the study is to examine associations of baseline residential elevation with blood biomarkers and newly reported multisystem chronic diseases among middle-aged and older adults, and to explore whether routine blood indicators may lie on these pathways. **Methods**: We analyzed 2011, 2015, and 2018 data from the China Health and Retirement Longitudinal Study (CHARLS). Wave 1 provided residential elevation, covariates, and 14 biomarkers; Wave 3 provided intermediate biomarkers; and Wave 4 identified 13 chronic disease outcomes based on participants’ reports of having received a physician diagnosis. High elevation was defined as ≥1500 m. Baseline and longitudinal samples included 11,371 and 9270 participants. Analyses used multivariable linear regression, attrition-weighted Cox models, logistic sensitivity models, restricted cubic splines, and exploratory mediation analysis. **Results**: High-elevation residence was associated with 9 of 14 biomarkers. Among 182 biomarker–disease Cox models, 35 associations had false-discovery-rate-adjusted q < 0.05, including 32 involving outcomes with at least 50 newly reported cases in the high-elevation group. Direct multiple-imputation inverse-probability-weighted (MI-IPW) Cox models showed that high-elevation residence was associated with higher estimated hazards over the interview interval for newly reported lung disease, kidney disease, arthritis, and hypertension; the first three associations remained significant in weighted logistic models. MI-IPW sensitivity analyses retained 31 of the 32 event-reliable biomarker–disease findings. No indirect effect survived multiple-comparison correction, including after excluding participants who had reported the relevant disease by Wave 3. Among the eight outcomes examined using restricted cubic splines, only kidney disease showed nominal evidence of nonlinearity (*p* = 0.017). **Conclusions**: Baseline residential elevation was associated with distinct biomarker patterns and heterogeneous disease risks. Routine blood biomarkers were prospectively associated with later disease reporting but did not provide stable evidence of mediation. These observational findings support cautious, disease-specific interpretation and further prospective and mechanistic research.

## 1. Introduction

The most prominent feature of high-altitude environments is chronic hypobaric hypoxia. It is estimated that approximately 500.3 million people worldwide live at elevations of ≥1500 m, including approximately 81.6 million who reside at elevations of ≥2500 m [1]. Traditionally, high-altitude medicine has focused primarily on acute illnesses caused by short-term exposure to high altitude, such as acute mountain sickness, high-altitude pulmonary edema, and high-altitude cerebral edema [2,3]. With accelerating population aging, increasing attention has been directed toward the long-term effects of high-altitude exposure on the development and progression of noncommunicable chronic diseases among middle-aged and older adults [4,5,6,7]. Clarifying the associations between high-altitude residence and chronic disease risk, as well as the potential pathways underlying these associations, is therefore important for environmental epidemiology and chronic disease prevention in high-altitude regions.

Epidemiological evidence regarding the health effects of long-term high-altitude residence remains inconsistent [8,9,10]. Some cross-sectional and ecological studies have suggested that high-altitude residence may exert protective effects against certain metabolic or cardiovascular diseases through vascular and metabolic adaptations [10,11]. In contrast, a recent population-based cross-sectional study using data from two prospective cohorts reported that long-term high-altitude exposure was associated with accelerated biological aging and multidimensional aging-related changes [4]. Other research has suggested that some aging-related biomarkers associated with disease and DNA damage may decrease with increasing elevation [5]. Cardiometabolic studies have also indicated that the association between residential elevation and estimated cardiovascular risk may vary according to individual metabolic status [12]. These findings suggest that the health effects of high-altitude exposure may not be uniformly beneficial or harmful, but may differ according to the disease system, elevation range, and characteristics of the study population. Previous studies have generally focused on single disease outcomes, cross-sectional comparisons, or ecological designs, whereas prospective comparisons across multiple chronic disease systems and continuous elevation gradients remain limited.

Chronic hypobaric hypoxia may affect erythropoiesis and systemic oxygenation [13,14], sympathetic and vascular regulation [7,15,16,17,18], and renal metabolism, purine metabolism, inflammatory responses, and lipid metabolism [19,20,21,22]. Routine blood biomarkers may therefore provide a practical means of characterizing physiological responses associated with residential elevation. However, biomarkers that vary across elevation levels are not necessarily those associated with later disease reporting. Likewise, a statistical association between a biomarker and a disease outcome is insufficient to establish that the biomarker mediates the relationship between elevation and disease. It is therefore necessary to distinguish clearly among three types of relationships in both analysis and interpretation: elevation–biomarker associations, biomarker–disease associations, and direct elevation–disease associations. In recent years, epidemiological studies have used mediation-analysis frameworks to investigate socioeconomic, metabolic, and environmental pathways [23,24,25,26], but such analyses do not in themselves establish causal mediation. Restricted cubic splines can be used to characterize potentially nonlinear elevation–risk relationships [27], whereas temporally ordered mediation models can be used to explore possible intermediate pathways. Importantly, causal interpretation of mediation effects in observational studies depends on several assumptions that are difficult to verify fully, including the absence of unmeasured confounding, correct temporal ordering, and appropriate model specification. The corresponding findings should therefore be regarded as exploratory evidence [28,29,30].

Using data from the 2011, 2015, and 2018 waves of the China Health and Retirement Longitudinal Study (CHARLS), we constructed a Wave 1–Wave 3–Wave 4 temporal framework linking baseline residential elevation, blood biomarkers measured at Wave 3, and new self-reports at Wave 4 of having received a physician diagnosis of a chronic disease. First, we examined the cross-sectional associations between high-altitude residence and 14 baseline blood biomarkers. We then evaluated the associations between baseline biomarkers and 13 subsequent disease outcomes and estimated the direct associations between residential elevation and these disease outcomes. Finally, we assessed the relationships between continuous elevation and disease risk and conducted exploratory mediation analyses using blood biomarkers measured at Wave 3. By distinguishing among these analytical components, this study aimed to determine whether, among middle-aged and older Chinese adults, routine blood biomarkers reflect physiological differences associated with residential elevation, are prospectively associated with subsequently reported chronic diseases, and provide exploratory evidence for potential intermediate pathways linking elevation to disease.

## 2. Materials and Methods

### 2.1. Study Design and Population

This prospective cohort analysis used Wave 1 (2011), Wave 3 (2015), and Wave 4 (2018) of the China Health and Retirement Longitudinal Study (CHARLS); Wave 2 was not used in the present analyses. CHARLS was designed as a nationally representative survey of middle-aged and older Chinese adults. However, the present analyses did not incorporate the original CHARLS survey design weights and should not be interpreted as nationally weighted population estimates. Wave 1 provided baseline residential elevation, covariates, and blood biomarkers; Wave 3 provided intermediate biomarkers; and Wave 4 provided later disease status. The exploratory mediation component was reported with reference to the AGReMA short-form guidance [30], and overall reporting was informed by STROBE recommendations [31]. The assumed directed acyclic graph is shown in Supplementary Figure S2.

**Figure 1:**
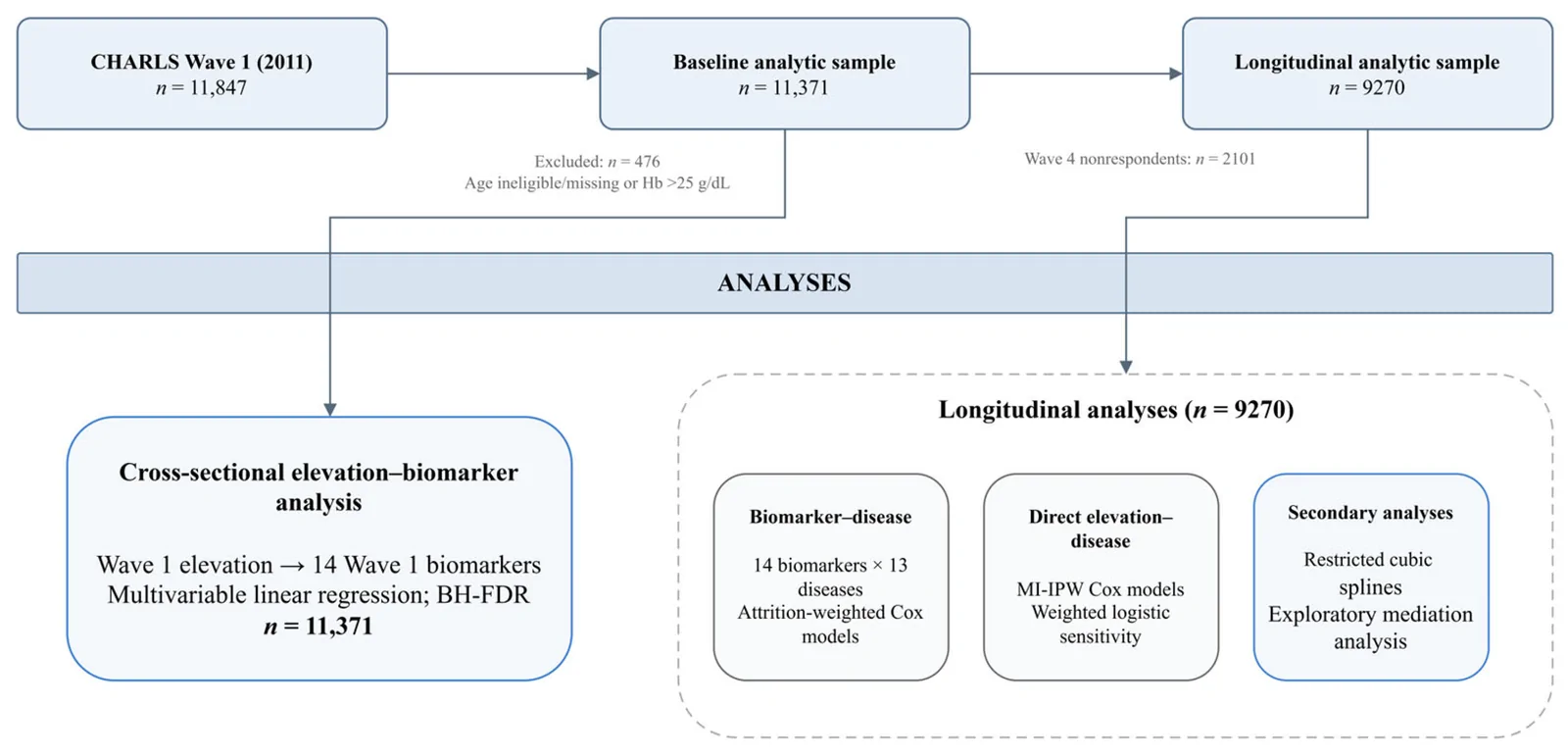
Study participant selection and analytical framework. Participants from CHARLS Wave 1 were screened to construct the baseline cross-sectional and longitudinal analytic samples. For each disease-specific analysis, participants who reported the corresponding diagnosis at Wave 1 were excluded. Wave 4 outcomes were defined as new participant reports of having received a physician diagnosis rather than as clinically adjudicated incident disease. Because exact onset dates were unavailable, Cox estimates represented approximate time-to-event associations based on interview intervals. BH-FDR, Benjamini–Hochberg false discovery rate; CHARLS, China Health and Retirement Longitudinal Study; Hb, hemoglobin; MI-IPW, multiple-imputation inverse-probability weighting.

Participants were eligible if they were aged 45 years or older at Wave 1. After excluding participants with missing or ineligible age information and those with physiologically implausible hemoglobin values, we constructed the baseline cross-sectional sample for the elevation–biomarker analysis. Participants without Wave 4 follow-up were then excluded to define the longitudinal sample for the biomarker–disease, direct elevation–disease, dose–response, and exploratory mediation analyses. Participant flow is shown in Figure 1, and detailed exclusion rules and disease-specific sample sizes are provided in Supplementary Tables S2 and S5.

### 2.2. Altitude Exposure, Biomarkers, and Outcomes

Baseline residential elevation was assigned by linking CHARLS community-level Global Positioning System coordinates to geographic elevation data. Because detailed information on residential duration, migration history, individual mobility, occupational altitude or hypoxia exposure, and indoor oxygenation conditions was unavailable in the analytic data, this measure represents residential elevation at baseline and was treated as a proxy for sustained altitude exposure rather than as a direct measure of lifetime hypoxic exposure. Elevation was analyzed both continuously and categorically. The ≥1500 m cutoff was prespecified and is consistent with a commonly used altitude classification in which 1500–2500 m is considered moderate altitude [8,10], and has also been applied in a previous CHARLS-based study [9]. Residence at ≥1500 m was classified as high altitude and residence at <1500 m as low altitude. Because altitude classifications vary across disciplines, sensitivity analyses repeated the direct elevation–disease models using thresholds of ≥2000 m and ≥2500 m.

Blood biomarkers were selected according to prespecified missingness and collinearity rules to avoid post hoc selection bias. Biomarkers with >20% missingness were excluded, and when two candidates were highly correlated (Pearson |r| > 0.85), the biomarker with lower completeness was excluded; fasting glucose was replaced by HbA1c, and cystatin C was excluded for excessive missingness. The final W1 biomarker panel included hemoglobin, hematocrit, white blood cell count, mean corpuscular volume, platelet count, triglycerides, high-density lipoprotein cholesterol, low-density lipoprotein cholesterol, total cholesterol, glycated hemoglobin, uric acid, C-reactive protein, blood urea nitrogen, and creatinine. The full biomarker screening process is shown in Supplementary Table S1.

Chronic disease outcomes were based on participants’ self-reports of physician diagnoses across CHARLS waves. For each outcome, the disease-specific longitudinal sample excluded participants who reported the corresponding diagnosis at Wave 1. A newly reported disease was defined as no self-report at Wave 1 of having received a physician diagnosis for the condition and a corresponding self-report at Wave 4. This operational definition does not denote clinically adjudicated incident disease. The prespecified outcomes were hypertension, dyslipidemia, diabetes, heart disease, stroke, lung disease, kidney disease, arthritis, liver disease, digestive disease, asthma, memory problem, and psychiatric problem. Because exact dates of diagnosis were unavailable, follow-up time was calculated from the Wave 1 to Wave 4 interview dates, and Cox estimates were interpreted as approximate time-to-event associations over the interview interval rather than associations with precisely observed onset times. Outcomes with fewer than 50 newly reported cases in the high-altitude group were considered low reliability and were excluded from the main interpretation.

### 2.3. Covariates and Missing Data

All substantive elevation–biomarker, biomarker–disease, and elevation–disease outcome models included Wave 1 age (continuous), sex (male or female), marital status (married/cohabiting or other), education (less than lower secondary, upper secondary or vocational, or tertiary), residence (rural or urban), body mass index (BMI; continuous), current smoking (yes or no), alcohol use (yes or no), and annual individual earnings transformed as ln(earnings + 1). Biomarker–disease models additionally adjusted for elevation group.

All retained biomarkers were screened for missingness and range. Participants with physiologically implausible hemoglobin values > 25 g/dL were excluded (*n* = 46) during sample construction. Triglycerides > 500 mg/dL (*n* = 135) were top-coded at 500 mg/dL to limit the leverage of extreme values; triglycerides < 10 mg/dL (*n* = 2), BMI ≤ 0 or >60 kg/m^2^ (*n* = 9), and Wave 1 MCV < 40 or >150 fL (*n* = 10) were set to missing. Detailed rules are provided in Supplementary Table S2.

Baseline biomarker missingness ranged from 0.07% to 2.73%; BMI missingness was 15.87%, and other covariates had ≤2.35% missingness (Supplementary Table S13). Missing BMI was imputed using multiple imputation by chained equations (MICE) with predictive mean matching in 20 datasets; Wave 1 biomarkers were not imputed in cross-sectional or Cox analyses. Mediation analyses used extended MICE for BMI and Wave 3 biomarkers, with corresponding Wave 1 values as auxiliary predictors. Estimates were pooled using Rubin’s Rules.

### 2.4. Statistical Analysis

Baseline characteristics were summarized by altitude group using means and standard deviations for continuous variables and counts and percentages for categorical variables. Standardized mean differences were used to describe between-group imbalance.

Cross-sectional associations between high-altitude residence and W1 blood biomarkers were evaluated using multivariable linear regression, with each biomarker modeled separately as the outcome. Models adjusted for baseline covariates, and estimates from imputed datasets were pooled using Rubin’s Rules. Standardized beta coefficients were reported to facilitate comparison across biomarkers. *p* values were corrected using the Benjamini–Hochberg false discovery rate (BH-FDR), with q < 0.05 considered statistically significant.

Associations between each Wave 1 biomarker and each newly reported disease were estimated using stabilized attrition-weighted Cox proportional hazards models. Models adjusted for age, sex, marital status, education, rural residence, BMI, current smoking, alcohol use, log-transformed annual individual earnings, and elevation group, and used community-clustered robust standard errors. Cox regression was retained as the prespecified primary model because participant-specific interview dates provided individual follow-up intervals, while recognizing that actual disease onset was observed only within survey intervals. Stabilized weights were estimated from a prespecified Wave 4 participation model that included 15 baseline blood measures—the 14 study biomarkers plus fasting glucose used only as an auxiliary predictor—eight baseline covariates, and 13 baseline disease indicators. The model was originally fitted among participants with complete data for all participation-model predictors, and weights were truncated at the 1st and 99th percentiles. Nominally significant predictors from the Wave 3 and Wave 4 attrition-screening models are reported in Supplementary Table S3, and differences between participants excluded from and included in the complete-case participation model are summarized in Supplementary Table S4. Participants whose complete-case participation weights could not be calculated because of missing participation-model predictors were retained with unit weights in the primary implementation to preserve the full Wave 4 analytic cohort and avoid excluding participants solely because of missing weight-model predictors. A unit weight indicated that no additional attrition correction was applied to these participants; it was not a model-derived estimate of their participation probability. The calculable attrition weights were not CHARLS survey sampling weights.

Cox-model *p* values were jointly corrected across the 182 biomarker–disease models using the Benjamini–Hochberg false discovery rate. Weighted logistic regression with the same covariates, attrition weights, and community-clustered standard errors was used as an alternative model that did not rely on exact event timing. Weighting sensitivity analyses included complete-weight cases and multiple-imputation inverse-probability weighting (MI-IPW), in which missing attrition-model predictors were imputed and imputation-specific stabilized weights were estimated for all Wave 4 participants. In biomarker-scale sensitivity analyses, TG, CRP, UA, and creatinine were natural-log transformed and standardized, whereas the remaining biomarkers were standardized without transformation. Proportional hazards assumptions were assessed using exposure-specific and global Schoenfeld-residual tests. Detailed sensitivity analyses and weight diagnostics are provided in Supplementary Tables S11 and S15.

Direct associations of high- versus low-elevation residence with each of the 13 newly reported diseases were estimated using MI-IPW Cox proportional hazards models adjusted for the prespecified baseline covariates and community-clustered robust standard errors. Weighted logistic regression was fitted using the same exposure definition and covariates, the primary attrition-weight implementation, and community-clustered robust standard errors as an alternative model that did not rely on exact event timing. Within each model family, *p* values for the 13 direct elevation–disease associations were corrected using the Benjamini–Hochberg false discovery rate. Alternative binary elevation thresholds of 2000 and 2500 m were evaluated in sensitivity analyses using Cox and weighted logistic regression models. These alternative-threshold models used the primary attrition-weight implementation at each cut point (Supplementary Table S14).

Exploratory mediation analyses used Wave 1 residential elevation as the exposure, Wave 3 biomarkers as candidate intermediate variables, and Wave 4 newly reported diseases as outcomes. Candidate pathways were required to be FDR-significant in the primary Cox analysis, to involve an outcome with at least 50 high-altitude cases, and to avoid diagnostic circularity. TG, HDL-C, LDL-C, and total cholesterol with dyslipidemia and creatinine with kidney disease were therefore excluded. HbA1c with diabetes was retained because participants had no self-reported physician diagnosis of diabetes at Wave 1 and HbA1c was modeled continuously. Model-based average causal mediation effects (ACMEs; retained as the standard estimand label) and average direct effects (ADEs) were estimated as absolute differences in model-estimated outcome probability comparing high- with low-elevation residence and pooled across 20 imputed datasets. Despite the conventional estimand terminology, all mediation estimates were interpreted as exploratory rather than as evidence of causality because the required no-unmeasured-confounding and temporal-ordering assumptions could not be verified. Multiplicity was evaluated using a Bonferroni-equivalent threshold of 0.05/27 (approximately 0.0019). To assess mediator–outcome temporality, all 27 candidate pathways were re-evaluated after excluding participants who had reported the corresponding disease by Wave 3. Complete primary mediation results are provided in Supplementary Table S8. Supplementary Table S12 presents the two pathways with nominal ACME *p* < 0.05 in the temporal-order sensitivity analysis; none of the 27 pathways remained significant after BH-FDR or Bonferroni correction.

For the HbA1c–diabetes association, a separate sensitivity analysis excluded participants with baseline HbA1c ≥ 6.5% despite no self-reported physician diagnosis of diabetes at Wave 1.

Effect modification was explored for the 32 FDR-significant and event-reliable biomarker–disease Cox associations across sex, age (<60 vs. ≥60 years), and residence (rural vs. urban), yielding 96 interaction tests. These exploratory analyses were not corrected for multiple comparisons. Dose–response associations between continuous baseline residential elevation and eight selected outcomes—hypertension, heart disease, stroke, lung disease, dyslipidemia, diabetes, arthritis, and kidney disease—were evaluated using restricted cubic spline Cox models. Four outcome-specific knots were placed at the 5th, 35th, 65th, and 95th percentiles of the disease-specific elevation distribution. Models were fitted across 20 imputed datasets using MI-IPW, adjustment for the prespecified baseline covariates, and community-clustered robust covariance estimates. Spline coefficients and covariance matrices were pooled across imputations, and nonlinearity was assessed using pooled Wald tests of the nonlinear spline terms. Hazard ratios were referenced to 0 m.

The primary biomarker–disease Cox models, biomarker-screening rules, disease set, 1500 m exposure threshold, and multiplicity-control framework were prespecified. Mediation and subgroup analyses were exploratory, and the restricted cubic spline models provided an exploratory assessment of nonlinearity. Complete-weight-case, MI-IPW, weighted logistic, alternative-threshold, biomarker-scale, baseline-HbA1c-exclusion, and temporal-order analyses were treated as post hoc sensitivity analyses.

All analyses were performed using R version 4.4.0 (R Foundation for Statistical Computing, Vienna, Austria). The principal R packages used were mice (version 3.19.0), survival (version 3.8.6), mediation (version 4.5.1), rms (version 8.1.1), sandwich (version 3.1.1), ggplot2 (version 4.0.3), and patchwork (version 1.3.2). A two-sided nominal significance level of 0.05 was used unless otherwise specified.

## 3. Results

### 3.1. Baseline Characteristics

The original CHARLS Wave 1 sample included 11,847 participants. After excluding participants aged <45 years or with missing age information and those with physiologically implausible hemoglobin values, 11,371 participants remained in the baseline cross-sectional analytic sample, including 1040 high-altitude residents and 10,331 low-altitude residents. Continuous residential elevation ranged from 1 to 4263 m. Among the 1040 participants in the baseline high-altitude group, the median residential elevation was 2252 m (interquartile range, 1855–2618 m); 368 participants resided at 1500–1999 m, 226 at 2000–2499 m, and 446 at ≥2500 m. After excluding Wave 4 non-respondents, the longitudinal analytic sample included 9270 participants, of whom 863 lived at high altitude and 8407 lived at low altitude. Follow-up ranged from 6.33 to 7.25 years. Participant flow is shown in Figure 1, and disease-specific analytic samples are provided in Supplementary Table S5.

Baseline characteristics are summarized in Table 1. Compared with low-altitude residents, high-altitude residents were more likely to live in rural areas and to have less than lower secondary education, and they had lower BMI and annual individual earnings. Differences in sex, smoking, and alcohol use were small. High-altitude residents had higher hemoglobin and mean corpuscular volume, lower white blood cell and platelet counts, generally lower HDL-C, LDL-C, and total cholesterol, and slightly higher triglycerides and blood urea nitrogen. Differences in HbA1c, uric acid, C-reactive protein, and creatinine were small.

The Wave 1–Wave 4 attrition rate was 18.5%, and primary attrition weights were calculable for 7238 participants. Before versus after 1st–99th percentile truncation, the mean (SD), range, and effective sample size (ESS) were 0.976 (0.137), 0.835–2.707, and 7098 versus 0.973 (0.120), 0.854–1.511, and 7130. After assigning unit weights to the remaining 2032 participants, the corresponding values were 0.979 (0.107), 0.854–1.511, and 9162. MI-IPW generated finite weights for all 9270 participants; across 20 imputations, the mean weight, mean SD, and mean ESS were 0.995, 0.143, and 9082, respectively (Supplementary Table S15).

### 3.2. Cross-Sectional Elevation–Biomarker Associations

**Figure 2:**
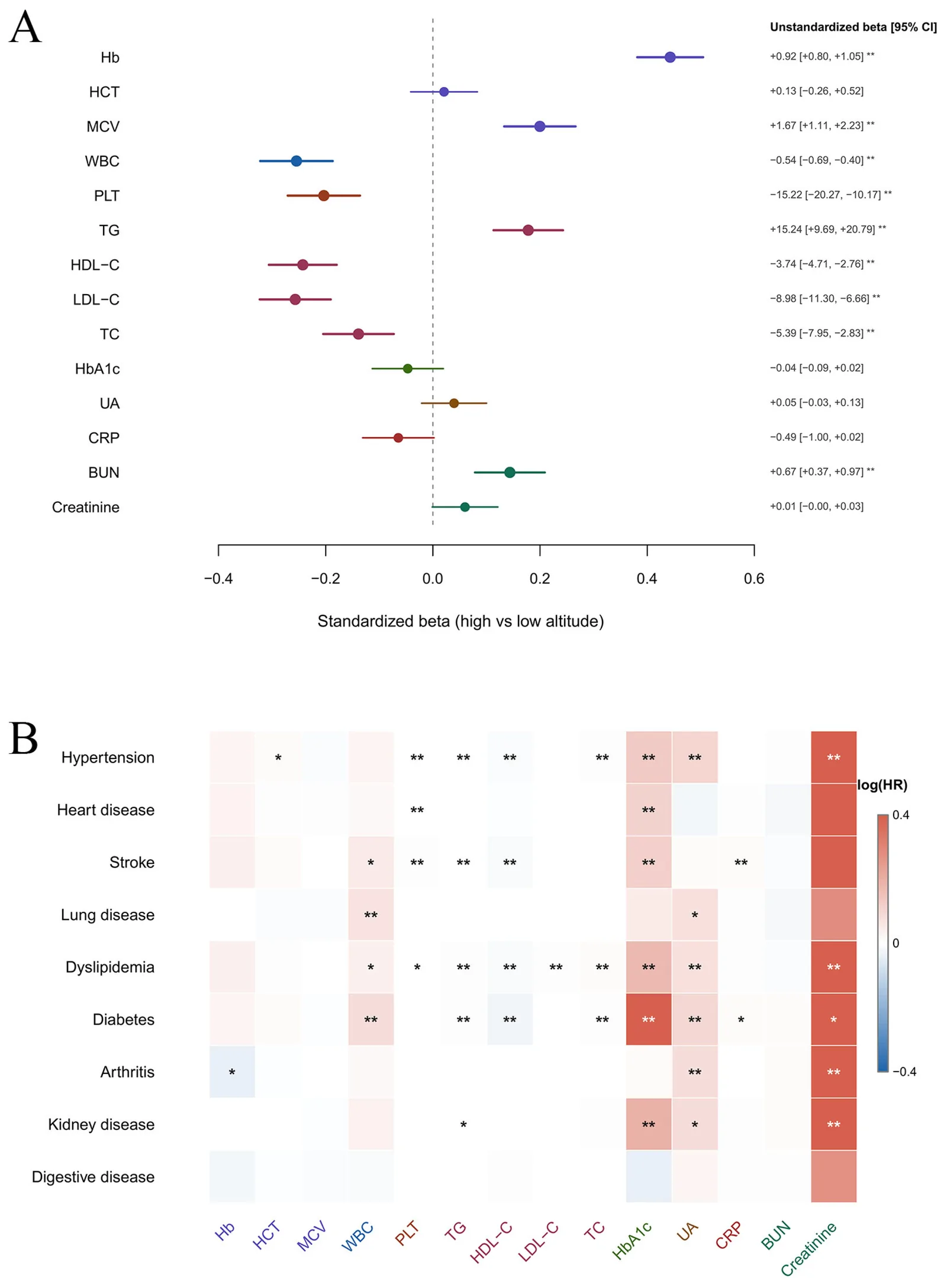
Cross-sectional elevation–biomarker associations and longitudinal biomarker–disease associations. (**A**) Adjusted differences in 14 Wave 1 biomarkers for residence at ≥1500 versus <1500 m. Points and horizontal lines show standardized β coefficients and 95% confidence intervals, and the right column shows unstandardized differences in the original biomarker units. (**B**) log(HR) values from the primary attrition-weighted Cox models for outcomes with at least 50 newly reported cases in the high-elevation group. HRs represent a one-unit increase: g/dL for Hb; percentage points for HCT and HbA1c; fL for MCV; ×10^9^/L for WBC and PLT; mg/dL for TG, HDL-C, LDL-C, TC, UA, BUN, and creatinine; and mg/L for CRP. Models adjusted for the prespecified baseline covariates; Panel (**B**) additionally adjusted for elevation group and used community-clustered robust standard errors. The Panel (**B**) color scale was truncated to the range −0.4 to 0.4 on the log(HR) scale; estimates outside this range are displayed using the endpoint colors. * *p* < 0.05; ** BH-FDR q < 0.05. CI, confidence interval; HR, hazard ratio. In Panel (**A**), colors are used only to distinguish individual biomarkers and do not encode additional statistical information; in Panel (**B**), the color scale represents log(HR), with warmer colors indicating positive associations and cooler colors indicating inverse associations.

After adjustment for demographic, socioeconomic, and lifestyle covariates, 9 of 14 Wave 1 blood biomarkers remained significantly associated with high-altitude residence after BH-FDR correction (Figure 2A). Hemoglobin, mean corpuscular volume, triglycerides, and blood urea nitrogen were higher in the high-altitude group, whereas white blood cell count, platelet count, HDL-C, LDL-C, and total cholesterol were lower. The largest adjusted differences were observed for hemoglobin (+0.925 g/dL), mean corpuscular volume (+1.673 fL), platelet count (−15.22 × 10^9^/L), triglycerides (+15.24 mg/dL), and LDL-C (−8.98 mg/dL). In contrast, hematocrit showed only a small and non-significant difference, and creatinine, uric acid, HbA1c, and C-reactive protein did not differ significantly after adjustment. Additional descriptive patterns for hemoglobin and hematocrit, including their joint distribution and the distribution of the Hb-to-HCT ratio, are shown in Supplementary Figure S1.

### 3.3. Direct Elevation–Disease and Biomarker–Disease Associations

Direct MI-IPW Cox models showed higher estimated hazards over the interview interval for newly reported lung disease (HR = 2.004; 95% CI, 1.493–2.689), kidney disease (HR = 1.965; 95% CI, 1.460–2.644), arthritis (HR = 1.764; 95% CI, 1.297–2.399), and hypertension (HR = 1.475; 95% CI, 1.126–1.931) among high- compared with low-elevation residents; all four associations had FDR-adjusted q < 0.05. Lung disease, kidney disease, and arthritis remained FDR-significant in weighted logistic models, whereas hypertension did not. In alternative-threshold analyses, kidney disease remained FDR-significant in both the Cox and weighted logistic models at the 2000 and 2500 m thresholds, whereas lung disease remained significant in both models at 2000 m but not at 2500 m. At the 2500 m threshold, an inverse association with diabetes was observed only in the weighted logistic model (OR = 0.570; 95% CI, 0.387–0.839; q = 0.029), whereas the corresponding Cox association did not meet the FDR significance threshold (Supplementary Table S14). Full estimates for all 13 outcomes are provided in Supplementary Table S10.

Among the 182 prespecified biomarker–disease Cox models, 35 associations met the FDR threshold, including 32 involving outcomes with at least 50 newly reported cases in the high-elevation group. The remaining three—CRP with asthma, WBC with psychiatric problems, and TG with liver disease—were considered exploratory because of low event counts. Weighted logistic models showed the same effect direction as the primary Cox models for 167 of 182 associations and retained 24 of the 32 event-reliable findings. MI-IPW estimates were directionally concordant with the primary Cox estimates for 180 of 182 associations and retained 34 of the 35 primary FDR-significant associations. Among the 32 event-reliable primary findings, 31 remained FDR-significant. The only change in FDR classification among the primary FDR-significant associations was the creatinine–dyslipidemia association, which remained positive but moved slightly above the FDR threshold (q = 0.061). Thus, the principal biomarker–disease conclusions were unchanged in the MI-IPW analysis. After biomarker transformation and standardization, 178 of 182 associations retained the same direction and 32 of the 35 primary FDR-significant findings remained significant. For TG specifically, all 13 TG–disease associations retained the same direction, and all five primary FDR-significant TG associations remained FDR-significant after natural-log transformation and standardization. No proportional-hazards test remained significant after FDR correction (Supplementary Table S11).

The biomarkers most consistently associated with later disease reporting were HbA1c, HDL-C, uric acid, creatinine, WBC, platelet count, and CRP (Figure 2B and Supplementary Table S6). HbA1c showed the broadest positive association pattern, with the strongest association observed for diabetes (HR = 1.866 per 1-percentage-point increase). After excluding participants with baseline HbA1c ≥ 6.5%, the HbA1c–diabetes association remained strong in both the Cox model (HR = 3.549; 95% CI, 2.853–4.417) and the weighted logistic model (OR = 3.805; 95% CI, 3.009–4.812) (Supplementary Table S11). Elevation-associated biomarkers only partially overlapped with biomarkers associated with later disease reporting: hemoglobin, mean corpuscular volume, and blood urea nitrogen showed clear elevation-related differences but were not broadly associated with later disease reporting, whereas HbA1c, uric acid, and creatinine were associated with several later-reported outcomes despite limited elevation-related differences. Because the biomarker–disease models were estimated in the overall cohort, this comparison is descriptive and should not be interpreted as evidence of an altitude-specific mechanism.

### 3.4. Exploratory Mediation Analysis

Among the 32 FDR-significant and event-reliable biomarker–disease associations, five diagnostically circular combinations were excluded, leaving 27 pathways for exploratory mediation analysis. No ACME met the Bonferroni-equivalent threshold of approximately 0.0019. Diabetes–WBC and diabetes–TG were nominally significant, with ACMEs of 0.0024 and 0.0014, respectively, but neither survived correction for multiple comparisons. After excluding participants who had reported the corresponding disease by Wave 3, both pathways remained nominally significant only, with a BH-adjusted q value of 0.616 for each; none of the 27 pathways survived BH-FDR or Bonferroni correction. Complete primary mediation results are provided in Supplementary Table S8, while Supplementary Table S12 presents the two nominal temporal-order sensitivity findings. The assumed directed acyclic graph is shown in Supplementary Figure S2.

### 3.5. Subgroup Analysis

Effect modification was assessed for the 32 FDR-significant and event-reliable Cox associations across sex, age (<60 vs. ≥60 years), and residence (rural vs. urban), yielding 96 interaction tests. Seven interaction terms reached nominal *p* < 0.05, but these analyses were exploratory and were not corrected for multiple comparisons. The relatively clearer patterns were a stronger association between HbA1c and kidney disease among participants aged <60 years and a stronger association between white blood cell count and diabetes among women. Residence-stratified results were particularly uncertain because the high-altitude urban sample was small. Complete subgroup interaction results are provided in Supplementary Table S7.

### 3.6. Dose–Response Relationships Between Elevation and Chronic Disease Risk

**Figure 3:**
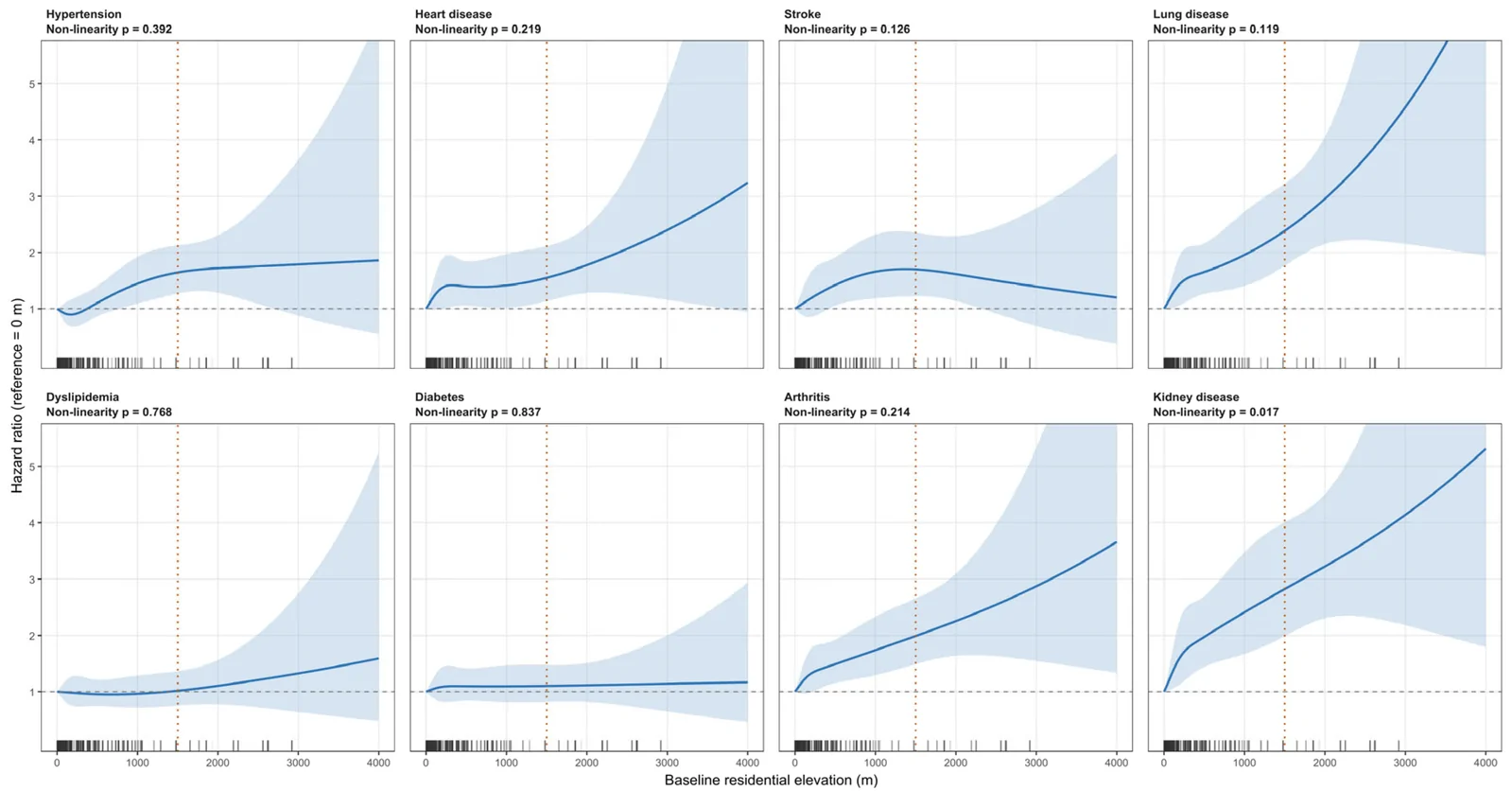
Restricted cubic spline dose–response associations between continuous baseline residential elevation and newly reported chronic diseases. Curves show pooled MI-IPW Cox hazard ratios and 95% confidence intervals from 20 imputed datasets, adjusted for the prespecified baseline covariates with community-clustered robust covariance estimates. Four outcome-specific knots were placed at the 5th, 35th, 65th, and 95th percentiles, and hazard ratios were referenced to 0 m. The orange dotted line marks the 1500 m binary classification threshold, and rug marks show the observed elevation distribution. Panel *p* values test nonlinearity. The displayed *y*-axis is limited to hazard ratios of 0.2–5.5; estimates at sparsely represented high elevations are imprecise. MI-IPW, multiple-imputation inverse-probability weighting.

Among the eight outcomes examined using pooled MI-IPW restricted cubic splines, only kidney disease showed nominal evidence of nonlinearity (*p* = 0.017); no other outcome had a nominally significant nonlinearity test (Supplementary Table S9 and Figure 3). Hazard ratios were referenced to 0 m, whereas 1500 m was only the binary classification threshold used in the primary analysis. Confidence intervals widened at sparsely represented high elevations, and estimates at the extreme upper end of the elevation distribution should therefore be interpreted cautiously. These spline models evaluated direct elevation–disease associations and were separate from the exploratory mediation analyses.

## 4. Discussion

In this cohort of middle-aged and older Chinese adults, baseline residential elevation was associated with distinct blood biomarker profiles and heterogeneous disease risks. High-elevation residence was associated with 9 of 14 biomarkers, while 35 of 182 biomarker–disease Cox models met the false-discovery-rate threshold, including 32 involving outcomes with at least 50 newly reported cases in the high-elevation group. Direct MI-IPW Cox models indicated higher estimated hazards over the interview interval for newly reported lung disease, kidney disease, arthritis, and hypertension, although only the first three were supported by weighted logistic regression. Biomarkers associated with elevation only partially overlapped with those associated with later disease reporting. No exploratory mediation pathway survived correction for multiple comparisons, and only kidney disease showed nominal evidence of nonlinearity. These findings concern three distinct analytical levels—elevation–biomarker, biomarker–disease, and direct elevation–disease associations—and should not be interpreted as an established elevation–biomarker–disease mechanism.

The direct elevation–disease findings varied across disease systems. Kidney disease showed the broadest consistency across threshold and dose–response analyses: it was FDR-significant in the primary 1500 m MI-IPW Cox model, remained FDR-significant in sensitivity analyses using 2000 and 2500 m thresholds, and was the only outcome with nominal evidence of nonlinearity. This pattern has biological plausibility because renal oxygenation depends on the balance between oxygen delivery and the high metabolic demands of the renal tubules, and previous studies have linked high-altitude or hypoxic exposure with altered renal function, hyperuricemia, and kidney injury [11,19,20,32,33,34,35,36]. A possible J-shaped association between hemoglobin and chronic kidney disease in a Tibetan population further suggests a complex relationship between hematological adaptation and kidney health [37]. However, these studies provide biological context only and do not establish that hypoxia or hematological adaptation caused the observed association. Moreover, widening confidence intervals at sparsely represented high elevations preclude interpreting the spline as a definitive threshold or causal dose–response relationship. Evidence for the other outcomes was less consistent. An inverse association with diabetes emerged only in the weighted logistic model at the 2500 m threshold and was not reproduced in the corresponding Cox model or at the lower elevation thresholds. This isolated, model-specific finding should therefore be regarded as exploratory. Previous respiratory studies have reported physiological effects of altitude but mixed associations with COPD prevalence and progression, together with greater underdiagnosis at higher elevations [38,39,40]. If underdiagnosis was more common among high-altitude residents, positive associations with self-reported lung disease would generally be attenuated, although the overall direction of ascertainment bias remains uncertain. Hypertension showed substantial heterogeneity across previous studies and was not supported by the logistic sensitivity model in the present analysis; this finding should therefore be interpreted cautiously [7,41,42,43]. The association with the broad self-reported arthritis outcome should likewise remain exploratory because prior evidence mainly concerned specific arthritis phenotypes and was largely cross-sectional or prevalence-based [44,45,46].

The biomarker findings also require separation of cross-sectional and longitudinal interpretations. HbA1c was associated with the broadest range of subsequently reported diseases despite little difference between elevation groups. Its association with diabetes remained strong after excluding participants with baseline HbA1c ≥ 6.5%, reducing concern that the result was driven entirely by undiagnosed diabetes at baseline. Nevertheless, altitude-related erythropoiesis and erythrocyte turnover may affect the relationship between HbA1c and glycemic status [14,47,48,49,50], so HbA1c should be interpreted here as a disease-risk biomarker rather than direct evidence of altitude-related glycemic change. High-elevation residents also had lower HDL-C, LDL-C, and total cholesterol but higher triglycerides. Although hypoxia may influence lipid metabolism and lipid profiles vary across elevation levels [21,22,51,52], HDL concentration does not fully represent HDL function [53], and these cross-sectional differences should not be interpreted uniformly as beneficial. More broadly, hemoglobin, mean corpuscular volume, and blood urea nitrogen showed elevation-related differences but were not broadly associated with later disease reporting, whereas HbA1c, uric acid, and creatinine were associated with several later-reported outcomes despite limited elevation differences. Because the biomarker–disease models were estimated in the overall cohort, this partial overlap is descriptive and does not establish altitude-specific mechanisms.

The mediation analyses provided no stable evidence that the evaluated routine blood biomarkers lay on the pathway between baseline residential elevation and later disease reporting. The two nominal findings did not survive correction for multiple comparisons, and temporal-order sensitivity analyses excluding participants who had reported the corresponding disease by Wave 3 did not materially change the conclusion. However, because disease status was observed only at discrete survey waves, some Wave 3 biomarker values may have been influenced by subclinical or undiagnosed disease that developed before Wave 3. Reverse causation therefore cannot be excluded, even after participants who reported the corresponding disease by Wave 3 were excluded, and these biomarkers should be interpreted as intermediate correlates rather than established mediators. These null results do not exclude biological mediation; rather, the analyses did not identify a robust intermediate pathway after multiplicity correction.

Strengths of this study included the large longitudinal sample, systematic evaluation of 14 biomarkers and 13 disease outcomes, prespecified multiplicity control, attrition weighting, community-clustered inference, and sensitivity analyses using logistic regression, MI-IPW, alternative elevation thresholds, biomarker rescaling, and proportional-hazards diagnostics.

Several limitations remain. Baseline community elevation was only a proxy for sustained exposure because detailed residential duration and migration history were unavailable within the present analytic framework, while daily mobility, occupational altitude or hypoxia exposure, indoor oxygenation conditions, and individual-level hypoxic exposure were not measured in the analytic data. Disease outcomes were self-reported physician diagnoses, and exact onset dates were not observed. Greater underdiagnosis in remote high-altitude areas could attenuate positive associations, but variation by disease type, healthcare access, residence, and socioeconomic conditions makes the overall direction of bias uncertain. Cox estimates should therefore be interpreted as approximate time-to-event associations across interview intervals. Measured blood pressure, heart rate, oxygen saturation, medication use, physical activity, diet, healthcare access, ethnicity or genetic adaptation, and harmonized measures of the severity of coexisting baseline conditions were unavailable or incompletely captured, leaving residual confounding. CHARLS survey design weights were not used, so the estimates are not nationally weighted. In the primary attrition-weighting analysis, weights were calculable for 7238 participants, whereas 2032 participants with missing participation-model predictors were assigned unit weights. Because these participants received no model-based attrition correction, residual selection bias may remain if predictor missingness was related to participation, residential elevation, biomarker levels, or disease outcomes; the direction and magnitude of this potential bias are uncertain. MI-IPW generated finite weights for all 9270 participants and yielded highly concordant results, retaining 31 of the 32 event-reliable primary findings, which reduces but does not eliminate this concern. Finally, the mediation analyses relied on unverifiable assumptions of no unmeasured confounding, while mediator–outcome temporal ordering remained uncertain.

## 5. Conclusions

In this CHARLS-based cohort, residential community elevation at baseline was associated with differences in multiple blood biomarker levels, whereas its associations with newly reported chronic disease outcomes during follow-up varied by disease type. In the MI-IPW Cox models, compared with the low-altitude group, the high-altitude group showed higher estimated hazards over the interview interval for newly reported lung disease, kidney disease, arthritis, and hypertension; however, the hypertension association was not supported by the weighted logistic regression sensitivity analysis. Biomarkers associated with residential elevation only partially overlapped with those associated with subsequent disease outcomes. No exploratory mediation pathway remained statistically significant after correction for multiple comparisons, and only kidney disease showed nominal evidence of a nonlinear association with elevation. Given the observational design, reliance on self-reported disease outcomes, and limited exposure information, these findings should be regarded as hypothesis-generating and require further validation in prospective studies incorporating repeated disease assessments, detailed residential histories, and individual-level measurements of hypoxic exposure.

## Figures and Tables

**Table 1:** Baseline characteristics of study participants by altitude group in the baseline cross-sectional analytic sample.

Baseline Characteristic	Low Altitude (<1500 m), *n* = 10,331	High Altitude (≥1500 m), *n* = 1040	SMD
Age, years, mean ± SD	59.19 ± 9.48	58.46 ± 9.22	0.078
Male, *n* (%)	4884 (47.3%)	488 (46.9%)	0.007
Married/cohabiting, *n* (%)	8552 (82.8%)	822 (79.0%)	0.095
**Education, *n* (%)**			0.160
— Less than lower secondary	9221 (89.3%)	974 (93.7%)	
— Upper secondary or vocational	960 (9.3%)	55 (5.3%)	
— Tertiary	150 (1.5%)	11 (1.1%)	
Rural residence, *n* (%)	8247 (79.9%)	935 (90.0%)	0.285
BMI, kg/m^2^, mean ± SD	23.61 ± 3.87	22.33 ± 3.71	0.337
Current smoking, *n* (%)	3000 (29.7%)	305 (30.5%)	0.018
Alcohol use, *n* (%)	4038 (39.3%)	391 (38.0%)	0.027
Log-transformed annual individual earnings, mean ± SD	1.84 ± 3.71	1.34 ± 3.17	0.144
**Blood biomarkers, mean ± SD**			
Hemoglobin (Hb), g/dL	14.28 ± 2.00	15.15 ± 2.75	0.365
Hematocrit (HCT), %	41.49 ± 6.06	40.99 ± 7.49	0.073
White blood cell count (WBC), ×10^9^/L	6.30 ± 2.15	5.75 ± 1.78	0.276
Mean corpuscular volume (MCV), fL	90.55 ± 8.36	92.27 ± 8.38	0.206
Platelet count (PLT), ×10^9^/L	213.56 ± 74.97	198.56 ± 71.56	0.205
Triglycerides (TG), mg/dL	130.37 ± 85.01	141.01 ± 90.72	0.121
High-density lipoprotein cholesterol (HDL-C), mg/dL	51.18 ± 15.55	48.66 ± 13.58	0.172
Low-density lipoprotein cholesterol (LDL-C), mg/dL	117.19 ± 34.94	107.28 ± 33.86	0.288
Total cholesterol (TC), mg/dL	193.88 ± 38.67	187.56 ± 39.92	0.161
Glycated hemoglobin (HbA1c), %	5.27 ± 0.83	5.22 ± 0.86	0.071
Uric acid (UA), mg/dL	4.48 ± 1.27	4.43 ± 1.27	0.041
C-reactive protein (CRP), mg/L	2.87 ± 7.85	2.32 ± 5.18	0.082
Blood urea nitrogen (BUN), mg/dL	15.74 ± 4.64	16.34 ± 4.75	0.128
Creatinine, mg/dL	0.79 ± 0.24	0.79 ± 0.21	0.006

Note. Data are presented as mean ± standard deviation or *n* (%). Low altitude was defined as residence at <1500 m and high altitude as residence at ≥1500 m. Standardized mean differences were used to describe between-group imbalance; SMD > 0.10 was considered to indicate meaningful imbalance. For education, the SMD represents the overall imbalance across the three-category variable. This table is based on the baseline cross-sectional sample of 11,371 participants. Missing BMI values were imputed using MICE-PMM for the adjusted cross-sectional analyses, whereas Wave 1 blood biomarkers were not imputed. BMI, body mass index; BUN, blood urea nitrogen; CRP, C-reactive protein; Hb, hemoglobin; HbA1c, glycated hemoglobin; HCT, hematocrit; HDL-C, high-density lipoprotein cholesterol; LDL-C, low-density lipoprotein cholesterol; MCV, mean corpuscular volume; MICE-PMM, multiple imputation by chained equations with predictive mean matching; PLT, platelet count; SD, standard deviation; SMD, standardized mean difference; TC, total cholesterol; TG, triglycerides; UA, uric acid; WBC, white blood cell count.

## Data Availability

The CHARLS data used in this study are available from the official CHARLS data platform (https://charls.charlsdata.com/pages/data/111/en.html (accessed on 21 June 2026)) upon registration and approval. Additional model-level results are available from the corresponding author upon reasonable request.

## Supplementary Materials

The following supporting information can be downloaded at https://www.mdpi.com/article/10.3390/healthcare14162554/s1. The Supplementary Materials include additional methodological details (Supplementary Methods); Figure S1: Hemoglobin and hematocrit patterns in the baseline cross-sectional analytic sample; Figure S2: Assumed directed acyclic graph for the exploratory mediation analysis; Table S1: Screening of candidate blood biomarkers at Wave 1; Table S2: Data preprocessing and exclusion rules; Table S3: Nominally significant predictors in Wave 3 and Wave 4 attrition-screening models; Table S4: Complete-case representativeness for stabilized inverse-probability weighting; Table S5: Disease-specific analytic sample sizes and newly reported case counts; Table S6: Complete weighted Cox regression results for 14 Wave 1 blood biomarkers and 13 newly reported chronic diseases; Table S7: Subgroup-specific associations and interaction tests for the 32 FDR-significant and event-reliable biomarker–disease associations; Table S8: Complete exploratory mediation results for the 27 candidate pathways selected using prespecified criteria; Table S9: Pooled restricted cubic spline nonlinearity tests for continuous elevation and newly reported chronic diseases; Table S10: Direct associations of high- versus low-elevation residence with newly reported disease outcomes; Table S11: Summary of sensitivity analyses. Table S12: Nominal ACME findings from the temporal-order sensitivity analysis after excluding participants who reported the corresponding disease by Wave 3. Table S13: Missingness of biomarkers and model covariates. Table S14: FDR-significant direct disease associations at the primary and alternative elevation thresholds using the primary attrition-weight implementation. Table S15: Diagnostics for attrition weights.

## References

[1] Tremblay J.C., Ainslie P.N. (2021). Global and Country-Level Estimates of Human Population at High Altitude. Proc. Natl. Acad. Sci. USA.

[2] Gatterer H., Villafuerte F.C., Ulrich S., Bhandari S.S., Keyes L.E., Burtscher M. (2024). Altitude Illnesses. Nat. Rev. Dis. Prim..

[3] Smedley T., Grocott M.P. (2013). Acute High-Altitude Illness: A Clinically Orientated Review. Br. J. Pain.

[4] Wu Y., Jin Y., Deng L., Wang Y., Wang Y., Chen J., Gao R., Wei S., Ni G., Zhou X. (2025). Long-Term High-Altitude Exposure, Accelerated Aging, and Multidimensional Aging-Related Changes. JAMA Netw. Open.

[5] Teklu A.A., Heckenbach I., Petr M.A., Bakula D., Keijzers G., Scheibye-Knudsen M. (2025). Deep Learning Reveals Diverging Effects of Altitude on Aging. GeroScience.

[6] Raberin A., Burtscher J., Burtscher M., Millet G.P. (2023). Hypoxia and the Aging Cardiovascular System. Aging Dis..

[7] Richalet J.P., Hermand E., Lhuissier F.J. (2024). Cardiovascular Physiology and Pathophysiology at High Altitude. Nat. Rev. Cardiol..

[8] Burtscher M. (2014). Effects of Living at Higher Altitudes on Mortality: A Narrative Review. Aging Dis..

[9] Wang S., Zhang F., Guo Y., Zhang C., Zhong Y., Hao D., Zhu S., Wu Y. (2025). Exposure to High Altitude Is Associated with an Elevated Risk of Hip Fracture: A Retrospective Cohort Study Using Data from the CHARLS. BMC Geriatr..

[10] Burtscher J., Samaja M. (2024). Healthy Aging at Moderate Altitudes: Hypoxia and Hormesis. Gerontology.

[11] Hurtado A., Escudero E., Pando J., Sharma S., Johnson R.J. (2012). Cardiovascular and Renal Effects of Chronic Exposure to High Altitude. Nephrol. Dial. Transplant..

[12] Xia Y., He L., Pang L., Zhu W., Zhang N., Gao Y., Duo L., Wang Z., Yang W., Tang M. (2025). Combined Effects of Insulin Resistance and Altitude of Residence on 10-Year Risk of Atherosclerotic Cardiovascular Disease. Front. Cardiovasc. Med..

[13] Guo Y., Li R., Laba C., Yan Q. (2026). U-shaped association between hematocrit and resting pulse oxygen saturation in Tibetan plateau population. Zhejiang Da Xue Xue Bao Yi Xue Ban..

[14] Zhou X., Bao Q., Cui Y., Li X., Yang C., Yang Y., Gao Y., Chen D., Huang J. (2025). Life Destiny of Erythrocyte in High Altitude Erythrocytosis: Mechanisms Underlying the Progression from Physiological (Moderate) to Pathological (Excessive) High-Altitude Erythrocytosis. Front. Genet..

[15] Swenson E.R. (2020). Sympathetic Nervous System Activation and Vascular Endothelial Function with Chronic Hypoxia. Circ. Res..

[16] Simpson L.L., Steinback C.D., Stembridge M., Moore J.P. (2021). A Sympathetic View of Blood Pressure Control at High Altitude: New Insights from Microneurographic Studies. Exp. Physiol..

[17] Simpson L.L., Stembridge M., Siebenmann C., Moore J.P., Lawley J.S. (2024). Mechanisms Underpinning Sympathoexcitation in Hypoxia. J. Physiol..

[18] Huang X., Akgün E.E., Mehmood K., Zhang H., Tang Z., Li Y. (2022). Mechanism of Hypoxia-Mediated Smooth Muscle Cell Proliferation Leading to Vascular Remodeling. BioMed Res. Int..

[19] Wang S.Y., Gao J., Zhao J.H. (2022). Effects of High Altitude on Renal Physiology and Kidney Diseases. Front. Physiol..

[20] Pu L., Xu H., Wang Z., Li R., Ai C., Song X., Zhang L., Cheng X., Wang G., Wang X. (2024). Intermittent High Altitude Hypoxia Induced Liver and Kidney Injury Leading to Hyperuricemia. Arch. Biochem. Biophys..

[21] Fan Y., Guo W., Yang L., Xu H. (2026). Effects and Adaptation of High-Altitude Hypoxia on Lipid Metabolism: Mechanisms and Health Implications. Front. Physiol..

[22] Bai X., Qi Y., Zhang Q. (2025). Dysregulation of Metabolites and High-Altitude Illnesses Development under Plateau Conditions. Front. Physiol..

[23] Yan C., Jing Y., Ruan Q., Liu P., Chen G. (2025). Socioeconomic Disparities in Physiological Dysregulation: A Longitudinal Mediation Analysis of Cardiometabolic Multimorbidity among Middle-Aged and Elderly Chinese. BMC Public Health.

[24] Wang P., Huang L., Zhu Z., Hu X., Wu B., Yang X. (2025). Mediation of Fasting Blood Glucose between Relative Muscle Strength and Hypertension: Insights from Two Cohorts. Diabetol. Metab. Syndr..

[25] Li Z., Wu W., Huang Y., Lawrence W.R., Lin S., Du Z., Wang Y., Hu S., Hao Y., Zhang W. (2024). Urban Residential Greenness and Cancer Mortality: Evaluating the Causal Mediation Role of Air Pollution in a Large Cohort. Environ. Pollut..

[26] Xu J., Yin T., Pan M., Qin L., Zhang L., Wang X., Zheng W., Liu C., Chen R. (2024). The Mediating Effect of TyG-Related Indicators between Long-Term Exposure to Particulate Matter and Cardiovascular Disease: Evidence from a National Longitudinal Cohort Study. Lipids Health Dis..

[27] Desquilbet L., Mariotti F. (2010). Dose-Response Analyses Using Restricted Cubic Spline Functions in Public Health Research. Stat. Med..

[28] Imai K., Keele L., Tingley D. (2010). A General Approach to Causal Mediation Analysis. Psychol. Methods.

[29] VanderWeele T.J. (2016). Mediation Analysis: A Practitioner’s Guide. Annu. Rev. Public Health.

[30] Lee H., Cashin A.G., Lamb S.E., Hopewell S., Vansteelandt S., VanderWeele T.J., MacKinnon D.P., Mansell G., Collins G.S., Golub R.M. (2021). A Guideline for Reporting Mediation Analyses of Randomized Trials and Observational Studies: The AGReMA Statement. JAMA.

[31] von Elm E., Altman D.G., Egger M., Pocock S.J., Gøtzsche P.C., Vandenbroucke J.P., STROBE Initiative (2007). The Strengthening the Reporting of Observational Studies in Epidemiology (STROBE) Statement: Guidelines for Reporting Observational Studies. PLoS Med..

[32] O’Connor P.M. (2006). Renal Oxygen Delivery: Matching Delivery to Metabolic Demand. Clin. Exp. Pharmacol. Physiol..

[33] Evans R.G., Smith D.W., Lee C.J., Ngo J.P., Gardiner B.S. (2020). What Makes the Kidney Susceptible to Hypoxia?. Anat. Rec..

[34] Zhang L., Feng E., Baima S., Laba C., Suolang L., Cang J., De Q. (2025). High Altitude Renal Syndrome: Four Elements or One Source?. Ren. Fail..

[35] Qiao R., Cui X., Hu Y., Wei H., Xu H., Zhang C., Du C., Chang J., Li Y., Ming W. (2024). Hypoxia Reduces Mouse Urine Output via HIF1α-Mediated Upregulation of Renal AQP1. Kidney Dis..

[36] Aoun M., Chelala D. (2022). Where Do You Live and What Do You Do? Two Questions That Might Impact Your Kidney Health. Front. Nephrol..

[37] Yin R., Li M., Liu C., Pu X., Yi W., Wu Y. (2025). A J-Shaped Relationship between Hemoglobin Levels and Chronic Kidney Disease. Sci. Rep..

[38] Titz A., Hoyos R., Ulrich S. (2024). Pulmonary Vascular Diseases at High Altitude—Is It Safe to Live in the Mountains?. Curr. Opin. Pulm. Med..

[39] Horner A., Soriano J.B., Puhan M.A., Studnicka M., Kaiser B., Vanfleteren L.E.G.W., Gnatiuc L., Burney P., Miravitlles M., García-Rio F. (2017). Altitude and COPD Prevalence: Analysis of the PREPOCOL-PLATINO-BOLD-EPI-SCAN Study. Respir. Res..

[40] Suri R., Markovic D., Woo H., Arjomandi M., Barr R.G., Bowler R.P., Criner G., Curtis J.L., Dransfield M.T., Drummond M.B. (2024). The Effect of Chronic Altitude Exposure on Chronic Obstructive Pulmonary Disease Outcomes in the SPIROMICS Cohort: An Observational Cohort Study. Am. J. Respir. Crit. Care Med..

[41] Aryal N., Weatherall M., Bhatta Y.K., Mann S. (2016). Blood Pressure and Hypertension in Adults Permanently Living at High Altitude: A Systematic Review and Meta-Analysis. High Alt. Med. Biol..

[42] Zhang X., Zhang Z., Ye R., Meng Q., Chen X. (2022). Prevalence of Hypertension and Its Relationship with Altitude in Highland Areas: A Systematic Review and Meta-Analysis. Hypertens. Res..

[43] Vinueza Veloz A.F., Yaulema Riss A.K., De Zeeuw C.I., Carpio Arias T.V., Vinueza Veloz M.F. (2020). Blood Pressure in Andean Adults Living Permanently at Different Altitudes. High Alt. Med. Biol..

[44] Zhang Q., Liu Q., Lin C., Baima Y., Li H., Gong H., Lin J. (2020). The Prevalence of Rheumatoid Arthritis in Middle-Aged and Elderly People Living in Naqu City, Tibet, Autonomous Region of China. J. Orthop. Surg. Res..

[45] Arima H., Koirala S., Nema K., Nakano M., Ito H., Poudel K.M., Pandey K., Pandey B.D., Yamamoto T. (2022). High Prevalence of Rheumatoid Arthritis and Its Risk Factors among Tibetan Highlanders Living in Tsarang, Mustang District of Nepal. J. Physiol. Anthropol..

[46] Chen X., Shao C., Li L., Zuo Z., Wang Y. (2025). Prevalence of Knee Osteoarthritis in the Chinese Population, 2013–2023: A Systematic Review and Meta-Analysis. J. Orthop. Surg. Res..

[47] Zheng R., Xu Y., Li M., Wang L., Lu J., Wang T., Xu M., Zhao Z., Zheng J., Dai M. (2024). Altitudes and Hemoglobin A1c Values: An Analysis Based on Two Nationwide Cross-Sectional Studies. Diabetes Care.

[48] Ren Q., Lv X., Yang L., Yue J., Luo Y., Zhou L., Meng S., Yang S., Puchi B., Zhou X. (2020). Erythrocytosis and Performance of HbA1c in Detecting Diabetes on an Oxygen-Deficient Plateau: A Population-Based Study. J. Clin. Endocrinol. Metab..

[49] Bazo-Alvarez J.C., Quispe R., Pillay T.D., Bernabé-Ortiz A., Smeeth L., Checkley W., Gilman R.H., Málaga G., Miranda J.J. (2017). Glycated Haemoglobin (HbA1c) and Fasting Plasma Glucose Relationships in Sea-Level and High-Altitude Settings. Diabet. Med..

[50] Sun Z., Wang S., He H., Zhang C., Li M., Ye Y., Zhang H., Yao X., Sun S., Du Y. (2025). Influence of High-Altitude Residential History on Optimal HbA1c Cutoff for Detecting Abnormal Glucose Metabolism. High Alt. Med. Biol..

[51] Alqahtani S.A.M. (2023). Lipid Profiles and Their Relation to Glycemic Control in Saudi Arabia: The Role of Altitudes and Environmental Factor. J. Fam. Med. Prim. Care.

[52] Ortiz-Prado E., Portilla D., Mosquera-Moscoso J., Simbaña-Rivera K., Duta D., Ochoa I., Burgos G., Izquierdo-Condoy J.S., Vásconez E., Calvopiña M. (2021). Hematological Parameters, Lipid Profile, and Cardiovascular Risk Analysis among Genotype-Controlled Indigenous Kiwcha Men and Women Living at Low and High Altitudes. Front. Physiol..

[53] Ouyang F.W., Chiang H.-H., Hsu W.-L., Tsai M.-H., Huang C.-Y., Remaley A.T., Akyol O., Chen C.-H. (2025). Dysfunctional High-Density Lipoprotein: An Updated Review. Front. Cardiovasc. Med..

