# Supplementary material for: High-Altitude Residence, Blood Biomarkers, and Chronic Disease Risk in Middle-Aged and Older Chinese Adults
Source: Healthcare (Basel). 2026 Aug 15;14(16):2554. doi: 10.3390/healthcare14162554 (PMC13512599; doi:10.3390/healthcare14162554)
Supplement: Supplementary file 1 [file healthcare-14-02554-s001.zip › healthcare-4425260-supplementary.pdf]

Supplementary Materials

Supplementary Materials for: High-Altitude Residence, Blood Biomarkers, and Chronic Disease Risk in Middle-Aged and Older Chinese Adults

This document provides extended methods, primary and sensitivity-analysis results, Supplementary Tables S1–S15, and Supplementary Figures S1–S2.

Supplementary Methods

1. Data preprocessing and construction of analytic samples

Triglycerides >500 mg/dL were top-coded at 500 mg/dL to limit the leverage of extreme values while retaining participants; values <10 mg/dL were set to missing. BMI values ≤0 or >60 kg/m² and Wave 1 MCV values <40 or >150 fL were set to missing before construction of the analytic samples. All retained biomarkers were screened for missingness, range, and collinearity according to the prespecified rules in Tables S1 and S2.

2. Blood biomarker screening

The missingness exclusion threshold was >20%, and the collinearity threshold for excluding a lower-completeness biomarker was a Pearson correlation of $|r|>0.85$ with another retained biomarker (see main-text Section 2.2 and Supplementary Table S1 for the full screening logic).

3. Missing data handling and multiple imputation

All imputation procedures used 20 datasets. Primary cross-sectional and Cox analyses imputed BMI only; baseline biomarkers used as study exposures or outcomes were not imputed. Mediation analyses used extended MICE for BMI and Wave 3 biomarkers. The MI-IPW sensitivity analysis additionally imputed missing predictors in the Wave 4 participation model. In the biomarker-scale sensitivity analysis, TG, CRP, UA, and creatinine were natural-log transformed and standardized; the remaining biomarkers were standardized without log transformation.

4. Attrition weighting and Cox model specification

The prespecified Wave 4 participation model included 15 baseline blood measures, comprising the 14 study biomarkers plus fasting glucose, which was used only as an auxiliary predictor in the participation model. The model also included eight baseline covariates and 13 baseline disease indicators.

The participation model was originally fitted among participants with complete data for all participation-model predictors. Stabilized weights were truncated at the 1st and 99th percentiles. To preserve the full Wave 4 analytic cohort and avoid excluding participants solely because of missing participation-model predictors, participants without calculable complete-case weights were assigned a unit weight in the primary implementation. A unit weight indicated that no additional model-based attrition correction was applied to these participants; it did not represent an estimated participation probability of 1. Because this pragmatic implementation could leave residual selection bias if predictor missingness was related to participation, residential elevation, biomarker levels, or disease outcomes, complete-weight-case and MI-IPW analyses were conducted as sensitivity analyses. In the MI-IPW analysis, missing participation-model predictors were imputed and imputation-specific stabilized weights were generated for all 9270 Wave 4 participants. Diagnostics before and after truncation, after unit-weight retention, and for MI-IPW are reported in Table S15. Weight means near 1 and high effective sample sizes indicated that no small subset dominated the weighted analyses, although residual selection bias cannot be excluded.

5. Candidate pathway selection for mediation analysis

The exploratory mediation analysis evaluated 27 candidate pathways selected using three prespecified eligibility criteria: BH-FDR $q < 0.05$ in the primary Cox analysis, at least 50 newly reported cases in the high-elevation group, and exclusion of diagnostically circular biomarker–disease pairs. For the temporal-order sensitivity analysis, all 27 candidate pathways were re-evaluated after excluding participants who had reported the corresponding disease by Wave 3.

Supplementary Table S12 presents the two pathways with nominal ACME $p < 0.05$; none of the 27 pathways remained significant after BH-FDR or Bonferroni correction.

6. Restricted cubic spline and subgroup analyses

RCS models provided an exploratory assessment of nonlinearity and used four outcome-specific knots at the 5th, 35th, 65th, and 95th elevation percentiles. Models were fitted in 20 imputed datasets using MI-IPW and community-clustered robust covariance estimates. Spline coefficients and covariance matrices were pooled, and nonlinearity was tested using pooled Wald tests. Subgroup analyses were restricted to the 32 FDR-significant and event-reliable biomarker-disease associations and were conducted across sex, age (<60 vs. ⩾60 years), and residence (rural vs. urban), yielding 96 interaction tests. The subgroup analyses were exploratory, and the 96 interaction tests were not corrected for multiple comparisons.

Supplementary Tables

Abbreviations used in Supplementary Tables and Figures

Abbreviation	Definition
ACME	Average causal mediation effect (standard estimand label)
ADE	Average direct effect
BH-FDR	Benjamini–Hochberg false discovery rate
BMI	Body mass index
BUN	Blood urea nitrogen
CHARLS	China Health and Retirement Longitudinal Study
CI	Confidence interval
CRP	C-reactive protein
DAG	Directed acyclic graph
FDR	False discovery rate
GLU	Fasting glucose
Hb	Hemoglobin
HbA1c	Glycated hemoglobin
HCT	Hematocrit
HDL-C	High-density lipoprotein cholesterol
HR	Hazard ratio
IPW	Inverse-probability weighting
LDL-C	Low-density lipoprotein cholesterol
MCV	Mean corpuscular volume
MICE-PMM	Multiple imputation by chained equations with predictive mean matching
OR	Odds ratio
PLT	Platelet count
RCS	Restricted cubic spline
SD	Standard deviation
SMD	Standardized mean difference
TC	Total cholesterol
TG	Triglycerides
UA	Uric acid
W1/W3/W4	CHARLS Waves 1, 3, and 4
WBC	White blood cell count
MI-IPW	Multiple-imputation inverse-probability weighting

Table S1. Screening of candidate blood biomarkers at Wave 1

Biomarker	W1 variable	Unit	Domain	Valid n	Missingness	Rule 1 passed	Rule 2 passed	Final status	Reason
Hb	r1Hemoglobin	g/dL	Erythrocyte-related	11070	2.6%	Yes	Yes	Included	Passed all prespecified screening criteria
HCT	r1Hematocrit	%	Erythrocyte-related	11363	0.1%	Yes	Yes	Included	Passed all prespecified screening criteria
WBC	r1WBC	×10⁹/L	Leukocyte-related	11067	2.7%	Yes	Yes	Included	Passed all prespecified screening criteria
MCV	r1MCV	fL	Erythrocyte-related	11061	2.7%	Yes	Yes	Included	Passed all prespecified screening criteria
PLT	r1Platelets	×10⁹/L	Platelet	11070	2.6%	Yes	Yes	Included	Passed all prespecified screening criteria
TG	r1tg	mg/dL	Lipid metabolism	11184	1.6%	Yes	Yes	Included	Passed all prespecified screening criteria
HDL-C	r1hdl	mg/dL	Lipid metabolism	11192	1.6%	Yes	Yes	Included	Passed all prespecified screening criteria

Biomarker	W1 variable	Unit	Domain	Valid n	Missingness	Rule 1 passed	Rule 2 passed	Final status	Reason
LDL-C	r1ldl	mg/dL	Lipid metabolism	11173	1.7%	Yes	Yes	Included	Passed all prespecified screening criteria
TC	r1cho	mg/dL	Lipid metabolism	11185	1.6%	Yes	Yes	Included	Passed all prespecified screening criteria
HbA1c	r1hba1c	%	Glucose metabolism	11236	1.2%	Yes	Yes	Included	Passed all prespecified screening criteria
GLU	r1glu	mg/dL	Glucose metabolism	11168	1.8%	Yes	No	Excluded	Prespecified decision: GLU was replaced by HbA1c
UA	r1ua	mg/dL	Purine metabolism	11193	1.6%	Yes	Yes	Included	Passed all prespecified screening criteria
CRP	r1crp	mg/L	Inflammation	11193	1.6%	Yes	Yes	Included	Passed all prespecified screening criteria
BUN	r1bun	mg/dL	Renal function	11191	1.6%	Yes	Yes	Included	Passed all prespecified screening criteria
Creatinine	r1crea	mg/dL	Renal function	11167	1.8%	Yes	Yes	Included	Passed all prespecified screening criteria
Cystatin C	r1Cystatin.C	mg/L	Renal function	8545	24.9%	No		Excluded	Rule 1: missingness exceeded the 20% threshold

Note. Candidate Wave 1 blood biomarkers were screened using prespecified missingness and collinearity rules. GLU was excluded from the 14-biomarker study panel by prespecified analytic decision and was replaced by HbA1c for the substantive biomarker analyses; however, GLU was retained as an auxiliary predictor in the Wave 4 participation model. Cystatin C was excluded because its missingness exceeded the 20% threshold.

Table S2. Data preprocessing and exclusion rules

Step	Procedure	Action	N before	N excluded/modified	N after	Note
1	Confirm W1 participation status	Confirmed inw1 = 1; the dataset had already been prefiltered and no additional exclusion was required.	11847	0	11847	Records with inw1 != 1: 0.
2	Exclude age <45 years or missing age	Removed participants aged <45 years; participants with missing age were also excluded.	11847	430	11417	430 participants were excluded, including 346 participants aged <45 years and 84 with missing age.
3	Exclude extreme hemoglobin values	Removed physiologically implausible hemoglobin values >25 g/dL.	11417	46	11371	46 participants were excluded; observed values ranged from 25.1 to 27.9 g/dL.
4	Process extreme triglyceride values without deleting participants	Top-coded TG values >500 mg/dL to 500 mg/dL and set TG values <10 mg/dL to missing.	11371	0	11371	135 values were top-coded and 2 values were set to missing; participant rows were unchanged.
5	Set extreme BMI values to missing	Set BMI values ≤0 or >60 kg/m2 to missing for subsequent MICE imputation.	11371	0	11371	9 values were set to missing and subsequently handled by MICE.
6	Set extreme W1 MCV values to missing	Set W1 MCV values <40 or >150 fL to missing.	11371	0	11371	10 values were set to missing.
7	Exclude Wave 4 non-respondents for longitudinal analyses	Restricted the longitudinal analytic sample to participants with Wave 4 outcome records.	11371	2101	9270	2101 participants were excluded; overall W1-to-W4 attrition was 18.5% (17.0% at high altitude and 18.6% at low altitude).

Note. N excluded/modified is reported as the number of participant rows excluded when rows were removed; for value-level corrections, participant rows were unchanged and the number indicates values modified or set to missing as documented in the source filtering report.

Table S3. Nominally significant predictors in Wave 3 and Wave 4 attrition-screening models

Wave	Predictor	OR	95% CI	*p* value	Direction
W3	BUN	0.971	0.954-0.988	<0.001	Lower attrition risk
W3	Creatinine	2.604	1.785-3.877	<0.001	Higher attrition risk
W3	CRP	1.017	1.010-1.024	<0.001	Higher attrition risk
W3	HbA1c	1.117	1.000-1.246	0.047	Higher attrition risk
W3	Hemoglobin	0.948	0.901-0.997	0.039	Lower attrition risk
W3	Age	1.032	1.023-1.040	<0.001	Higher attrition risk
W3	Sex (1=male, 2=female)	1.257	1.014-1.560	0.038	Higher attrition risk
W3	Rural residence	0.583	0.488-0.699	<0.001	Lower attrition risk
W3	Individual earnings (log)	1.000	1.000-1.000	0.012	Higher attrition risk
W3	Baseline lung disease	1.493	1.183-1.872	<0.001	Higher attrition risk
W3	Baseline diabetes	0.693	0.491-0.961	0.032	Lower attrition risk
W4	Fasting glucose	1.002	1.000-1.004	0.041	Higher attrition risk
W4	Creatinine	1.517	1.117-2.125	0.012	Higher attrition risk
W4	CRP	1.014	1.008-1.021	<0.001	Higher attrition risk
W4	Uric acid	1.092	1.033-1.154	0.002	Higher attrition risk
W4	Age	1.051	1.044-1.058	<0.001	Higher attrition risk
W4	BMI	0.961	0.943-0.979	<0.001	Lower attrition risk
W4	Rural residence	0.607	0.519-0.711	<0.001	Lower attrition risk
W4	Baseline lung disease	1.617	1.335-1.953	<0.001	Higher attrition risk
W4	Baseline memory problem	1.730	1.161-2.546	0.006	Higher attrition risk

Note. The complete-case attrition-bias screening models included 36 candidate predictors: 15 Wave 1 blood measures comprising the 14 study biomarkers plus fasting glucose; eight baseline covariates comprising age, sex, education, rural residence, BMI, current smoking, alcohol use, and log-transformed annual individual earnings; and 13 baseline disease indicators. Marital status was not included in the participation model. The table lists predictors with nominal $p < 0.05$. The final MI-IPW sensitivity model included all prespecified predictors regardless of nominal significance.

Table S4. Complete-case representativeness for stabilized inverse-probability weighting

Characteristic	Excluded from IPW complete-case model (*n* =2692)	Complete cases entering IPW model (*n* =8679)	*p* value	SMD
Age, years, mean (SD)	58.67 (9.94)	59.26 (9.29)	0.005	0.061
Sex (1=male, 2=female), mean (SD)	1.51 (0.50)	1.53 (0.50)	0.017	0.053
BMI, kg/m2, mean (SD)	23.58 (3.86)	23.49 (3.88)	0.480	0.025
Baseline hypertension, proportion (SD)	0.30 (0.46)	0.27 (0.44)	0.001	0.074
Baseline heart disease, proportion (SD)	0.14 (0.35)	0.12 (0.33)	0.018	0.052
Baseline stroke, proportion (SD)	0.04 (0.19)	0.03 (0.16)	<0.001	0.076
Baseline diabetes, proportion (SD)	0.07 (0.26)	0.06 (0.24)	0.026	0.049
Baseline lung disease, proportion (SD)	0.10 (0.30)	0.10 (0.30)	0.707	0.008
Low-altitude residence, *n* (%)	2428 (90.2%)	7903 (91.1%)	0.186	0.030

Note. Complete cases were participants with all predictors required by the original participation model. Among Wave 4 participants, 7238 had calculable complete-case participation weights, whereas 2032 participants with missing participation-model predictors were retained with unit weights to preserve the full Wave 4 analytic cohort. A unit weight indicated that no additional model-based attrition correction was applied and was not an estimated participation probability. The MI-IPW sensitivity analysis imputed missing participation-model predictors and generated finite weights for all 9270 Wave 4 participants.

Table S5. Disease-specific analytic sample sizes and newly reported case counts

Disease outcome	Baseline prevalent cases	Disease-specific analytic n	Newly reported cases by Wave 4	High-elevation newly reported cases	Low-elevation newly reported cases	Baseline prevalence	Event-reliable outcome
Asthma	381	8821	319	34	285	4.1%	No
Digestive disease	2095	7112	1129	111	1018	22.8%	Yes
Kidney disease	526	8648	604	77	527	5.7%	Yes
Dyslipidemia	907	8119	1457	102	1355	10.0%	Yes
Arthritis	3230	5984	1256	139	1117	35.1%	Yes
Psychiatric problem	127	9074	245	28	217	1.4%	No
Stroke	212	8995	658	52	606	2.3%	Yes

Disease outcome	Baseline prevalent cases	Disease-specific analytic n	Newly reported cases by Wave 4	High-elevation newly reported cases	Low-elevation newly reported cases	Baseline prevalence	Event-reliable outcome
Heart disease	1086	8093	1151	95	1056	11.8%	Yes
Lung disease	804	8393	983	127	856	8.7%	Yes
Diabetes	538	8603	834	59	775	5.9%	Yes
Hypertension	2404	6775	1752	173	1579	26.2%	Yes
Liver disease	309	8850	477	42	435	3.4%	No
Memory problem	117	9091	348	21	327	1.3%	No

Note. A newly reported disease was defined as no self-report at Wave 1 of having received a physician diagnosis for the condition and a corresponding self-report at Wave 4. This operational definition does not denote clinically adjudicated incident disease. Event-reliable outcomes were defined as those with at least 50 newly reported cases in the high-elevation group.

Table S6. Complete weighted Cox regression results for 14 Wave 1 blood biomarkers and 13 newly reported chronic diseases

Biomarker (unit; HR per 1-unit increase)	Disease outcome	HR	95% CI	*p*	*BH-FDR q*	Disease-eligible n	Newly reported cases in disease-eligible sample	High-altitude newly reported cases in disease-eligible sample	Reliable outcome	FDR significant
Hb (g/dL)	Asthma	1.046	0.991-1.105	0.100	0.313	8821	319	34	No	No
HCT (%)	Asthma	1.007	0.985-1.030	0.525	0.677	8821	319	34	No	No
WBC (×10⁹/L)	Asthma	1.077	1.010-1.148	0.025	0.107	8821	319	34	No	No
MCV (fL)	Asthma	0.997	0.981-1.014	0.725	0.829	8821	319	34	No	No
PLT (×10⁹/L)	Asthma	0.999	0.997-1.001	0.337	0.558	8821	319	34	No	No
TG (mg/dL)	Asthma	1.000	0.999-1.002	0.812	0.865	8821	319	34	No	No
HDL-C (mg/dL)	Asthma	1.007	0.998-1.015	0.111	0.327	8821	319	34	No	No
LDL-C (mg/dL)	Asthma	0.999	0.996-1.003	0.630	0.765	8821	319	34	No	No
TC (mg/dL)	Asthma	1.001	0.998-1.004	0.453	0.620	8821	319	34	No	No
HbA1c (%)	Asthma	1.052	0.931-1.190	0.414	0.608	8821	319	34	No	No
UA (mg/dL)	Asthma	1.061	0.959-1.174	0.249	0.496	8821	319	34	No	No
CRP (mg/L)	Asthma	1.013	1.003-1.022	0.008	0.043	8821	319	34	No	Yes
BUN	Asthma	1.005	0.978-1.033	0.706	0.829	8821	319	34	No	No

Biomarker (unit; HR per 1-unit increase)	Disease outcome	HR	95% CI	*p*	*BH-FDR q*	Disease-eligible n	Newly reported cases in disease-eligible sample	High-altitude newly reported cases in disease-eligible sample	Reliable outcome	FDR significant
(mg/dL)										
Creatinine (mg/dL)	Asthma	0.950	0.425-2.122	0.900	0.937	8821	319	34	No	No
Hb (g/dL)	Digestive disease	0.982	0.943-1.021	0.360	0.569	7112	1129	111	Yes	No
HCT (%)	Digestive disease	0.995	0.982-1.007	0.420	0.608	7112	1129	111	Yes	No
WBC (×10⁹/L)	Digestive disease	0.989	0.954-1.024	0.532	0.677	7112	1129	111	Yes	No
MCV (fL)	Digestive disease	0.993	0.984-1.003	0.149	0.377	7112	1129	111	Yes	No
PLT (×10⁹/L)	Digestive disease	1.000	0.999-1.001	0.611	0.746	7112	1129	111	Yes	No
TG (mg/dL)	Digestive disease	1.000	0.999-1.001	0.843	0.887	7112	1129	111	Yes	No
HDL-C (mg/dL)	Digestive disease	1.003	0.998-1.007	0.251	0.496	7112	1129	111	Yes	No
LDL-C (mg/dL)	Digestive disease	1.001	0.999-1.003	0.302	0.524	7112	1129	111	Yes	No
TC (mg/dL)	Digestive disease	1.001	1.000-1.003	0.149	0.377	7112	1129	111	Yes	No
HbA1c (%)	Digestive disease	0.959	0.888-1.037	0.294	0.515	7112	1129	111	Yes	No
UA (mg/dL)	Digestive disease	1.023	0.966-1.082	0.440	0.612	7112	1129	111	Yes	No
CRP (mg/L)	Digestive disease	1.004	0.997-1.010	0.313	0.529	7112	1129	111	Yes	No
BUN (mg/dL)	Digestive disease	1.003	0.987-1.019	0.738	0.830	7112	1129	111	Yes	No
Creatinine	Digestive disease	1.316	0.923-1.877	0.129	0.354	7112	1129	111	Yes	No

Biomarker (unit; HR per 1-unit increase)	Disease outcome	HR	95% CI	*p*	*BH-FDR q*	Disease-eligible n	Newly reported cases in disease-eligible sample	High-altitude newly reported cases in disease-eligible sample	Reliable outcome	FDR significant
(mg/dL)										
Hb (g/dL)	Kidney disease	0.999	0.952-1.049	0.975	0.986	8648	604	77	Yes	No
HCT (%)	Kidney disease	0.998	0.984-1.012	0.797	0.865	8648	604	77	Yes	No
WBC (×10⁹/L)	Kidney disease	1.034	0.984-1.087	0.185	0.427	8648	604	77	Yes	No
MCV (fL)	Kidney disease	0.998	0.987-1.009	0.718	0.829	8648	604	77	Yes	No
PLT (×10⁹/L)	Kidney disease	1.001	1.000-1.002	0.139	0.373	8648	604	77	Yes	No
TG (mg/dL)	Kidney disease	1.001	1.000-1.002	0.028	0.117	8648	604	77	Yes	No
HDL-C (mg/dL)	Kidney disease	1.001	0.995-1.007	0.764	0.850	8648	604	77	Yes	No
LDL-C (mg/dL)	Kidney disease	1.001	0.998-1.003	0.580	0.713	8648	604	77	Yes	No
TC (mg/dL)	Kidney disease	1.002	1.000-1.004	0.079	0.264	8648	604	77	Yes	No
HbA1c (%)	Kidney disease	1.217	1.119-1.323	<0.001	<0.001	8648	604	77	Yes	Yes
UA (mg/dL)	Kidney disease	1.090	1.008-1.178	0.030	0.121	8648	604	77	Yes	No
CRP (mg/L)	Kidney disease	1.003	0.994-1.013	0.517	0.672	8648	604	77	Yes	No
BUN (mg/dL)	Kidney disease	1.014	0.992-1.037	0.223	0.461	8648	604	77	Yes	No
Creatinine (mg/dL)	Kidney disease	4.630	2.941-7.289	<0.001	<0.001	8648	604	77	Yes	Yes
Hb (g/dL)	Dyslipidemia	1.035	0.999-1.073	0.060	0.217	8119	1457	102	Yes	No

Biomarker (unit; HR per 1-unit increase)	Disease outcome	HR	95% CI	*p*	*BH-FDR q*	Disease-eligible n	Newly reported cases in disease-eligible sample	High-altitude newly reported cases in disease-eligible sample	Reliable outcome	FDR significant
HCT (%)	Dyslipidemia	1.005	0.992-1.018	0.438	0.612	8119	1457	102	Yes	No
WBC (×10⁹/L)	Dyslipidemia	1.035	1.003-1.068	0.033	0.131	8119	1457	102	Yes	No
MCV (fL)	Dyslipidemia	0.998	0.989-1.007	0.684	0.814	8119	1457	102	Yes	No
PLT (×10⁹/L)	Dyslipidemia	1.001	1.000-1.001	0.044	0.169	8119	1457	102	Yes	No
TG (mg/dL)	Dyslipidemia	1.003	1.002-1.004	<0.001	<0.001	8119	1457	102	Yes	Yes
HDL-C (mg/dL)	Dyslipidemia	0.986	0.982-0.991	<0.001	<0.001	8119	1457	102	Yes	Yes
LDL-C (mg/dL)	Dyslipidemia	1.005	1.003-1.007	<0.001	<0.001	8119	1457	102	Yes	Yes
TC (mg/dL)	Dyslipidemia	1.006	1.005-1.008	<0.001	<0.001	8119	1457	102	Yes	Yes
HbA1c (%)	Dyslipidemia	1.194	1.133-1.258	<0.001	<0.001	8119	1457	102	Yes	Yes
UA (mg/dL)	Dyslipidemia	1.077	1.024-1.133	0.004	0.024	8119	1457	102	Yes	Yes
CRP (mg/L)	Dyslipidemia	1.004	0.997-1.011	0.288	0.510	8119	1457	102	Yes	No
BUN (mg/dL)	Dyslipidemia	0.991	0.977-1.005	0.197	0.438	8119	1457	102	Yes	No
Creatinine (mg/dL)	Dyslipidemia	1.606	1.136-2.270	0.007	0.040	8119	1457	102	Yes	Yes
Hb (g/dL)	Arthritis	0.959	0.928-0.992	0.016	0.071	5984	1256	139	Yes	No
HCT (%)	Arthritis	0.998	0.987-1.009	0.682	0.814	5984	1256	139	Yes	No
WBC	Arthritis	1.017	0.982-1.053	0.348	0.568	5984	1256	139	Yes	No

Biomarker (unit; HR per 1-unit increase)	**Disease outcome**	**HR**	**95% CI**	***p***	***BH-FDR q***	**Disease-eligible n**	**Newly reported cases in disease-eligible sample**	**High-altitude newly reported cases in disease-eligible sample**	**Reliable outcome**	**FDR significant**
(×10⁹/L)										
MCV (fL)	Arthritis	0.999	0.990-1.007	0.739	0.830	5984	1256	139	Yes	No
PLT (×10⁹/L)	Arthritis	1.000	0.999-1.001	0.498	0.658	5984	1256	139	Yes	No
TG (mg/dL)	Arthritis	1.000	1.000-1.001	0.421	0.608	5984	1256	139	Yes	No
HDL-C (mg/dL)	Arthritis	1.000	0.996-1.004	0.961	0.977	5984	1256	139	Yes	No
LDL-C (mg/dL)	Arthritis	1.001	0.999-1.003	0.335	0.558	5984	1256	139	Yes	No
TC (mg/dL)	Arthritis	1.001	1.000-1.003	0.072	0.253	5984	1256	139	Yes	No
HbA1c (%)	Arthritis	1.012	0.945-1.084	0.725	0.829	5984	1256	139	Yes	No
UA (mg/dL)	Arthritis	1.086	1.029-1.147	0.003	0.017	5984	1256	139	Yes	Yes
CRP (mg/L)	Arthritis	1.000	0.993-1.007	0.956	0.977	5984	1256	139	Yes	No
BUN (mg/dL)	Arthritis	1.011	0.996-1.026	0.156	0.383	5984	1256	139	Yes	No
Creatinine (mg/dL)	Arthritis	1.763	1.155-2.690	0.009	0.045	5984	1256	139	Yes	Yes
Hb (g/dL)	Psychiatric problem	1.015	0.953-1.081	0.635	0.765	9074	245	28	No	No
HCT (%)	Psychiatric problem	0.997	0.973-1.022	0.809	0.865	9074	245	28	No	No
WBC (×10⁹/L)	Psychiatric problem	0.901	0.836-0.972	0.007	0.040	9074	245	28	No	Yes
MCV (fL)	Psychiatric problem	1.010	0.993-1.028	0.250	0.496	9074	245	28	No	No

Biomarker (unit; HR per 1-unit increase)	Disease outcome	HR	95% CI	*p*	*BH-FDR q*	Disease-eligible n	Newly reported cases in disease-eligible sample	High-altitude newly reported cases in disease-eligible sample	Reliable outcome	FDR significant
PLT (×10⁹/L)	Psychiatric problem	0.999	0.997-1.000	0.124	0.354	9074	245	28	No	No
TG (mg/dL)	Psychiatric problem	1.001	0.999-1.002	0.428	0.608	9074	245	28	No	No
HDL-C (mg/dL)	Psychiatric problem	1.005	0.994-1.015	0.383	0.596	9074	245	28	No	No
LDL-C (mg/dL)	Psychiatric problem	0.999	0.996-1.003	0.766	0.850	9074	245	28	No	No
TC (mg/dL)	Psychiatric problem	1.001	0.998-1.004	0.571	0.708	9074	245	28	No	No
HbA1c (%)	Psychiatric problem	1.071	0.953-1.204	0.247	0.496	9074	245	28	No	No
UA (mg/dL)	Psychiatric problem	0.921	0.808-1.049	0.214	0.448	9074	245	28	No	No
CRP (mg/L)	Psychiatric problem	0.989	0.962-1.016	0.425	0.608	9074	245	28	No	No
BUN (mg/dL)	Psychiatric problem	1.009	0.980-1.038	0.556	0.698	9074	245	28	No	No
Creatinine (mg/dL)	Psychiatric problem	0.841	0.334-2.119	0.712	0.829	9074	245	28	No	No
Hb (g/dL)	Stroke	1.036	0.988-1.086	0.147	0.377	8995	658	52	Yes	No
HCT (%)	Stroke	1.011	0.994-1.028	0.200	0.439	8995	658	52	Yes	No
WBC (×10⁹/L)	Stroke	1.053	1.012-1.096	0.011	0.058	8995	658	52	Yes	No
MCV (fL)	Stroke	1.000	0.989-1.011	0.990	0.995	8995	658	52	Yes	No
PLT (×10⁹/L)	Stroke	1.002	1.001-1.003	<0.001	0.002	8995	658	52	Yes	Yes

Biomarker (unit; HR per 1-unit increase)	**Disease outcome**	**HR**	**95% CI**	***p***	***BH-FDR q***	**Disease-eligible n**	**Newly reported cases in disease-eligible sample**	**High-altitude newly reported cases in disease-eligible sample**	**Reliable outcome**	**FDR significant**
TG (mg/dL)	Stroke	1.002	1.001-1.003	<0.001	<0.001	8995	658	52	Yes	Yes
HDL-C (mg/dL)	Stroke	0.988	0.982-0.994	<0.001	<0.001	8995	658	52	Yes	Yes
LDL-C (mg/dL)	Stroke	1.001	0.998-1.003	0.489	0.654	8995	658	52	Yes	No
TC (mg/dL)	Stroke	1.001	0.999-1.004	0.184	0.427	8995	658	52	Yes	No
HbA1c (%)	Stroke	1.128	1.046-1.217	0.002	0.013	8995	658	52	Yes	Yes
UA (mg/dL)	Stroke	1.010	0.941-1.083	0.781	0.857	8995	658	52	Yes	No
CRP (mg/L)	Stroke	1.013	1.005-1.021	<0.001	0.007	8995	658	52	Yes	Yes
BUN (mg/dL)	Stroke	0.992	0.972-1.011	0.390	0.601	8995	658	52	Yes	No
Creatinine (mg/dL)	Stroke	1.509	0.961-2.370	0.074	0.253	8995	658	52	Yes	No
Hb (g/dL)	Heart disease	1.029	0.995-1.066	0.099	0.313	8093	1151	95	Yes	No
HCT (%)	Heart disease	1.005	0.993-1.017	0.411	0.608	8093	1151	95	Yes	No
WBC (×10⁹/L)	Heart disease	1.016	0.982-1.052	0.361	0.569	8093	1151	95	Yes	No
MCV (fL)	Heart disease	1.004	0.994-1.014	0.447	0.616	8093	1151	95	Yes	No
PLT (×10⁹/L)	Heart disease	1.001	1.000-1.002	0.003	0.017	8093	1151	95	Yes	Yes
TG (mg/dL)	Heart disease	1.000	1.000-1.001	0.457	0.621	8093	1151	95	Yes	No
HDL-C	Heart disease	0.998	0.994-1.002	0.357	0.569	8093	1151	95	Yes	No

Biomarker (unit; HR per 1-unit increase)	**Disease outcome**	**HR**	**95% CI**	***p***	***BH-FDR q***	**Disease-eligible n**	**Newly reported cases in disease-eligible sample**	**High-altitude newly reported cases in disease-eligible sample**	**Reliable outcome**	**FDR significant**
(mg/dL)										
LDL-C (mg/dL)	Heart disease	1.001	0.999-1.003	0.207	0.444	8093	1151	95	Yes	No
TC (mg/dL)	Heart disease	1.001	0.999-1.002	0.510	0.667	8093	1151	95	Yes	No
HbA1c (%)	Heart disease	1.121	1.049-1.197	<0.001	0.006	8093	1151	95	Yes	Yes
UA (mg/dL)	Heart disease	0.982	0.926-1.041	0.532	0.677	8093	1151	95	Yes	No
CRP (mg/L)	Heart disease	1.004	0.996-1.013	0.284	0.510	8093	1151	95	Yes	No
BUN (mg/dL)	Heart disease	0.986	0.970-1.002	0.085	0.276	8093	1151	95	Yes	No
Creatinine (mg/dL)	Heart disease	1.501	0.984-2.290	0.059	0.217	8093	1151	95	Yes	No
Hb (g/dL)	Lung disease	1.000	0.958-1.045	0.996	0.996	8393	983	127	Yes	No
HCT (%)	Lung disease	0.992	0.979-1.006	0.261	0.506	8393	983	127	Yes	No
WBC (×10⁹/L)	Lung disease	1.071	1.030-1.113	<0.001	0.005	8393	983	127	Yes	Yes
MCV (fL)	Lung disease	0.993	0.985-1.003	0.159	0.385	8393	983	127	Yes	No
PLT (×10⁹/L)	Lung disease	1.000	0.999-1.001	0.487	0.654	8393	983	127	Yes	No
TG (mg/dL)	Lung disease	1.001	1.000-1.001	0.139	0.373	8393	983	127	Yes	No
HDL-C (mg/dL)	Lung disease	1.001	0.996-1.006	0.818	0.865	8393	983	127	Yes	No
LDL-C	Lung disease	1.000	0.998-1.002	0.805	0.865	8393	983	127	Yes	No

Biomarker (unit; HR per 1-unit increase)	Disease outcome	HR	95% CI	*p*	*BH-FDR q*	Disease-eligible n	Newly reported cases in disease-eligible sample	High-altitude newly reported cases in disease-eligible sample	Reliable outcome	FDR significant
(mg/dL)										
TC (mg/dL)	Lung disease	1.001	0.999-1.003	0.289	0.510	8393	983	127	Yes	No
HbA1c (%)	Lung disease	1.048	0.977-1.125	0.191	0.434	8393	983	127	Yes	No
UA (mg/dL)	Lung disease	1.080	1.015-1.150	0.015	0.071	8393	983	127	Yes	No
CRP (mg/L)	Lung disease	1.005	0.998-1.012	0.195	0.438	8393	983	127	Yes	No
BUN (mg/dL)	Lung disease	0.986	0.971-1.001	0.072	0.253	8393	983	127	Yes	No
Creatinine (mg/dL)	Lung disease	1.335	0.898-1.984	0.153	0.382	8393	983	127	Yes	No
Hb (g/dL)	Diabetes	1.023	0.983-1.064	0.265	0.507	8603	834	59	Yes	No
HCT (%)	Diabetes	1.007	0.994-1.020	0.279	0.510	8603	834	59	Yes	No
WBC ($\times 10^9$/L)	Diabetes	1.087	1.052-1.123	<0.001	<0.001	8603	834	59	Yes	Yes
MCV (fL)	Diabetes	0.996	0.987-1.005	0.362	0.569	8603	834	59	Yes	No
PLT ($\times 10^9$/L)	Diabetes	1.001	1.000-1.002	0.278	0.510	8603	834	59	Yes	No
TG (mg/dL)	Diabetes	1.004	1.003-1.004	<0.001	<0.001	8603	834	59	Yes	Yes
HDL-C (mg/dL)	Diabetes	0.977	0.971-0.982	<0.001	<0.001	8603	834	59	Yes	Yes
LDL-C (mg/dL)	Diabetes	1.001	0.999-1.003	0.203	0.440	8603	834	59	Yes	No
TC (mg/dL)	Diabetes	1.004	1.002-1.006	<0.001	<0.001	8603	834	59	Yes	Yes

Biomarker (unit; HR per 1-unit increase)	**Disease outcome**	**HR**	**95% CI**	***p***	***BH-FDR q***	**Disease-eligible n**	**Newly reported cases in disease-eligible sample**	**High-altitude newly reported cases in disease-eligible sample**	**Reliable outcome**	**FDR significant**
HbA1c (%)	Diabetes	1.866	1.707-2.040	<0.001	<0.001	8603	834	59	Yes	Yes
UA (mg/dL)	Diabetes	1.107	1.038-1.182	0.002	0.014	8603	834	59	Yes	Yes
CRP (mg/L)	Diabetes	1.009	1.002-1.016	0.015	0.071	8603	834	59	Yes	No
BUN (mg/dL)	Diabetes	1.008	0.992-1.025	0.314	0.529	8603	834	59	Yes	No
Creatinine (mg/dL)	Diabetes	1.593	1.031-2.459	0.036	0.139	8603	834	59	Yes	No
Hb (g/dL)	Hypertension	1.025	0.995-1.057	0.103	0.313	6775	1752	173	Yes	No
HCT (%)	Hypertension	1.013	1.002-1.024	0.017	0.076	6775	1752	173	Yes	No
WBC (×10⁹/L)	Hypertension	1.026	0.997-1.056	0.080	0.264	6775	1752	173	Yes	No
MCV (fL)	Hypertension	0.994	0.986-1.002	0.128	0.354	6775	1752	173	Yes	No
PLT (×10⁹/L)	Hypertension	1.001	1.001-1.002	<0.001	0.002	6775	1752	173	Yes	Yes
TG (mg/dL)	Hypertension	1.002	1.001-1.002	<0.001	<0.001	6775	1752	173	Yes	Yes
HDL-C (mg/dL)	Hypertension	0.993	0.989-0.997	<0.001	0.007	6775	1752	173	Yes	Yes
LDL-C (mg/dL)	Hypertension	1.001	1.000-1.003	0.168	0.402	6775	1752	173	Yes	No
TC (mg/dL)	Hypertension	1.002	1.001-1.004	0.002	0.013	6775	1752	173	Yes	Yes
HbA1c (%)	Hypertension	1.141	1.082-1.202	<0.001	<0.001	6775	1752	173	Yes	Yes
UA (mg/dL)	Hypertension	1.110	1.058-1.164	<0.001	<0.001	6775	1752	173	Yes	Yes

Biomarker (unit; HR per 1-unit increase)	**Disease outcome**	**HR**	**95% CI**	***p***	***BH-FDR q***	**Disease-eligible n**	**Newly reported cases in disease-eligible sample**	**High-altitude newly reported cases in disease-eligible sample**	**Reliable outcome**	**FDR significant**
CRP (mg/L)	Hypertension	1.000	0.994-1.006	0.943	0.969	6775	1752	173	Yes	No
BUN (mg/dL)	Hypertension	1.004	0.991-1.016	0.572	0.708	6775	1752	173	Yes	No
Creatinine (mg/dL)	Hypertension	1.790	1.296-2.473	<0.001	0.004	6775	1752	173	Yes	Yes
Hb (g/dL)	Liver disease	1.026	0.976-1.078	0.312	0.529	8850	477	42	No	No
HCT (%)	Liver disease	1.006	0.990-1.023	0.438	0.612	8850	477	42	No	No
WBC (×10⁹/L)	Liver disease	0.978	0.928-1.031	0.409	0.608	8850	477	42	No	No
MCV (fL)	Liver disease	0.999	0.986-1.012	0.901	0.937	8850	477	42	No	No
PLT (×10⁹/L)	Liver disease	0.998	0.996-1.000	0.012	0.058	8850	477	42	No	No
TG (mg/dL)	Liver disease	1.002	1.001-1.003	<0.001	0.002	8850	477	42	No	Yes
HDL-C (mg/dL)	Liver disease	0.997	0.990-1.004	0.350	0.568	8850	477	42	No	No
LDL-C (mg/dL)	Liver disease	0.999	0.996-1.002	0.425	0.608	8850	477	42	No	No
TC (mg/dL)	Liver disease	1.002	0.999-1.004	0.181	0.427	8850	477	42	No	No
HbA1c (%)	Liver disease	1.043	0.922-1.180	0.499	0.658	8850	477	42	No	No
UA (mg/dL)	Liver disease	1.072	0.985-1.167	0.106	0.317	8850	477	42	No	No
CRP (mg/L)	Liver disease	0.995	0.983-1.007	0.396	0.605	8850	477	42	No	No
BUN	Liver disease	1.004	0.982-1.026	0.729	0.829	8850	477	42	No	No

Biomarker (unit; HR per 1-unit increase)	Disease outcome	HR	95% CI	*p*	*BH-FDR q*	Disease-eligible n	Newly reported cases in disease-eligible sample	High-altitude newly reported cases in disease-eligible sample	Reliable outcome	FDR significant
(mg/dL)										
Creatinine (mg/dL)	Liver disease	1.545	0.915-2.609	0.103	0.313	8850	477	42	No	No
Hb (g/dL)	Memory problem	1.034	0.974-1.098	0.274	0.510	9091	348	21	No	No
HCT (%)	Memory problem	1.003	0.981-1.025	0.817	0.865	9091	348	21	No	No
WBC (×10⁹/L)	Memory problem	1.061	1.006-1.118	0.028	0.117	9091	348	21	No	No
MCV (fL)	Memory problem	1.012	0.992-1.032	0.259	0.506	9091	348	21	No	No
PLT (×10⁹/L)	Memory problem	1.000	0.999-1.002	0.550	0.695	9091	348	21	No	No
TG (mg/dL)	Memory problem	1.001	1.000-1.002	0.124	0.354	9091	348	21	No	No
HDL-C (mg/dL)	Memory problem	0.996	0.988-1.004	0.288	0.510	9091	348	21	No	No
LDL-C (mg/dL)	Memory problem	1.002	0.999-1.006	0.211	0.446	9091	348	21	No	No
TC (mg/dL)	Memory problem	1.002	0.999-1.006	0.147	0.377	9091	348	21	No	No
HbA1c (%)	Memory problem	1.019	0.896-1.159	0.771	0.851	9091	348	21	No	No
UA (mg/dL)	Memory problem	1.019	0.923-1.124	0.715	0.829	9091	348	21	No	No
CRP (mg/L)	Memory problem	0.999	0.986-1.013	0.923	0.955	9091	348	21	No	No
BUN (mg/dL)	Memory problem	0.984	0.956-1.013	0.287	0.510	9091	348	21	No	No
Creatinine	Memory problem	1.265	0.707-2.262	0.427	0.608	9091	348	21	No	No

Biomarker (unit; HR per 1-unit increase)	Disease outcome	HR	95% CI	*p*	*BH-FDR q*	Disease-eligible n	Newly reported cases in disease-eligible sample	High-altitude newly reported cases in disease-eligible sample	Reliable outcome	FDR significant
(mg/dL)										

Note. This table contains all 182 biomarker–disease models. HRs correspond to continuous Wave 1 biomarkers in their original units. P values were corrected jointly across all 182 models using the Benjamini–Hochberg FDR procedure. Event-reliable outcomes were those with at least 50 newly reported cases in the high-elevation group. The three displayed sample-count columns are disease-level eligibility counts calculated after exclusion of baseline-prevalent cases but before biomarker- and covariate-specific complete-case exclusions; they are not the n and event counts of each fitted Cox model.

Table S7. Subgroup-specific associations and interaction tests for the 32 FDR-significant and event-reliable biomarker–disease associations

Biomarker	Disease outcome	Subgroup dimension	Subgroup-specific HRs (95% CI)	Interaction p value
HbA1c	Kidney disease	Sex	Male: 1.158 (1.006–1.332); Female: 1.271 (1.158–1.394)	0.291
HbA1c	Kidney disease	Age	<60: 1.339 (1.223–1.465); ≥60: 1.047 (0.889–1.233)	**0.009**
HbA1c	Kidney disease	Residence	Rural: 1.221 (1.114–1.339); Urban: 1.211 (1.000–1.468)	0.868
Creatinine	Kidney disease	Sex	Male: 6.537 (3.212–13.306); Female: 3.261 (1.674–6.354)	0.163
Creatinine	Kidney disease	Age	<60: 3.219 (1.796–5.770); ≥60: 7.143 (3.185–16.021)	0.143
Creatinine	Kidney disease	Residence	Rural: 4.269 (2.679–6.802); Urban: 7.202 (2.049–25.314)	0.906
TG	Dyslipidemia	Sex	Male: 1.003 (1.002–1.004); Female: 1.003 (1.002–1.004)	0.971
TG	Dyslipidemia	Age	<60: 1.003 (1.003–1.004); ≥60: 1.003 (1.002–1.004)	0.166
TG	Dyslipidemia	Residence	Rural: 1.003 (1.002–1.004); Urban: 1.003 (1.002–1.004)	0.574
HDL-C	Dyslipidemia	Sex	Male: 0.986 (0.981–0.992); Female: 0.986 (0.980–0.992)	0.910
HDL-C	Dyslipidemia	Age	<60: 0.985 (0.980–0.991); ≥60: 0.988 (0.981–0.994)	0.596
HDL-C	Dyslipidemia	Residence	Rural: 0.986 (0.981–0.991); Urban: 0.988 (0.979–0.997)	0.249
LDL-C	Dyslipidemia	Sex	Male: 1.007 (1.004–1.010); Female: 1.004 (1.002–1.006)	**0.029**
LDL-C	Dyslipidemia	Age	<60: 1.004 (1.002–1.006); ≥60: 1.006 (1.004–1.009)	0.218
LDL-C	Dyslipidemia	Residence	Rural: 1.006 (1.004–1.007); Urban: 1.003 (1.000–1.006)	0.282
TC	Dyslipidemia	Sex	Male: 1.007 (1.005–1.009); Female: 1.006 (1.004–1.007)	0.200
TC	Dyslipidemia	Age	<60: 1.006 (1.005–1.008); ≥60: 1.006 (1.004–1.008)	0.551
TC	Dyslipidemia	Residence	Rural: 1.007 (1.005–1.008); Urban: 1.005 (1.002–1.008)	0.400
HbA1c	Dyslipidemia	Sex	Male: 1.244 (1.147–1.349); Female: 1.169 (1.092–1.252)	0.226
HbA1c	Dyslipidemia	Age	<60: 1.187 (1.106–1.275); ≥60: 1.199 (1.105–1.300)	0.958
HbA1c	Dyslipidemia	Residence	Rural: 1.205 (1.140–1.274); Urban: 1.132 (0.984–1.303)	0.490
UA	Dyslipidemia	Sex	Male: 1.087 (1.014–1.165); Female: 1.068 (0.996–1.145)	0.566
UA	Dyslipidemia	Age	<60: 1.124 (1.059–1.194); ≥60: 1.019 (0.940–1.105)	0.126
UA	Dyslipidemia	Residence	Rural: 1.067 (1.009–1.129); Urban: 1.098 (0.986–1.223)	0.885
Creatinine	Dyslipidemia	Sex	Male: 1.644 (1.055–2.563); Female: 1.531 (0.936–2.503)	0.837
Creatinine	Dyslipidemia	Age	<60: 1.819 (1.140–2.901); ≥60: 1.357 (0.832–2.213)	0.860
Creatinine	Dyslipidemia	Residence	Rural: 1.339 (0.888–2.021); Urban: 2.715 (1.396–5.278)	0.113
UA	Arthritis	Sex	Male: 1.083 (1.005–1.167); Female: 1.094 (1.013–1.182)	0.812
UA	Arthritis	Age	<60: 1.085 (1.011–1.164); ≥60: 1.090 (1.008–1.179)	0.601
UA	Arthritis	Residence	Rural: 1.080 (1.020–1.144); Urban: 1.119 (0.981–1.277)	0.599
Creatinine	Arthritis	Sex	Male: 2.369 (1.467–3.827); Female: 1.177 (0.621–2.230)	0.088
Creatinine	Arthritis	Age	<60: 1.659 (0.972–2.832); ≥60: 1.777 (1.016–3.107)	0.477
Creatinine	Arthritis	Residence	Rural: 1.774 (1.084–2.904); Urban: 1.710 (0.797–3.670)	0.917
PLT	Stroke	Sex	Male: 1.002 (1.001–1.004); Female: 1.002 (1.001–1.004)	0.951
PLT	Stroke	Age	<60: 1.002 (1.001–1.004); ≥60: 1.002 (1.000–1.003)	0.754
PLT	Stroke	Residence	Rural: 1.002 (1.001–1.003); Urban: 1.003 (1.000–1.006)	0.531
TG	Stroke	Sex	Male: 1.002 (1.001–1.003); Female: 1.002 (1.001–1.003)	0.600
TG	Stroke	Age	<60: 1.001 (1.000–1.003); ≥60: 1.002 (1.001–1.003)	0.582
TG	Stroke	Residence	Rural: 1.002 (1.001–1.003); Urban: 1.002 (1.000–1.003)	0.611
HDL-C	Stroke	Sex	Male: 0.988 (0.980–0.996); Female: 0.988 (0.979–0.996)	0.880
HDL-C	Stroke	Age	<60: 0.988 (0.979–0.997); ≥60: 0.988 (0.980–0.996)	0.534
HDL-C	Stroke	Residence	Rural: 0.988 (0.981–0.995); Urban: 0.987 (0.972–1.002)	0.570
HbA1c	Stroke	Sex	Male: 1.085 (0.960–1.227); Female: 1.152 (1.045–1.270)	0.530

Biomarker	Disease outcome	Subgroup dimension	Subgroup-specific HRs (95% CI)	Interaction p value
HbA1c	Stroke	Age	<60: 1.172 (1.060–1.296); ≥60: 1.084 (0.970–1.211)	0.364
HbA1c	Stroke	Residence	Rural: 1.106 (1.009–1.212); Urban: 1.206 (1.039–1.401)	0.412
CRP	Stroke	Sex	Male: 1.011 (1.001–1.021); Female: 1.015 (1.002–1.028)	0.611
CRP	Stroke	Age	<60: 1.014 (1.004–1.024); ≥60: 1.012 (1.001–1.023)	0.864
CRP	Stroke	Residence	Rural: 1.009 (1.000–1.018); Urban: 1.028 (1.014–1.043)	**0.020**
PLT	Heart disease	Sex	Male: 1.001 (0.999–1.002); Female: 1.001 (1.001–1.002)	0.407
PLT	Heart disease	Age	<60: 1.001 (1.000–1.002); ≥60: 1.001 (1.000–1.003)	0.892
PLT	Heart disease	Residence	Rural: 1.001 (1.000–1.002); Urban: 1.002 (1.000–1.004)	0.185
HbA1c	Heart disease	Sex	Male: 1.062 (0.943–1.197); Female: 1.149 (1.064–1.241)	0.237
HbA1c	Heart disease	Age	<60: 1.163 (1.076–1.258); ≥60: 1.072 (0.962–1.195)	0.185
HbA1c	Heart disease	Residence	Rural: 1.136 (1.056–1.222); Urban: 1.061 (0.910–1.237)	0.539
WBC	Lung disease	Sex	Male: 1.069 (1.014–1.127); Female: 1.071 (1.020–1.124)	0.975
WBC	Lung disease	Age	<60: 1.063 (1.011–1.119); ≥60: 1.083 (1.026–1.144)	0.708
WBC	Lung disease	Residence	Rural: 1.061 (1.018–1.106); Urban: 1.127 (1.023–1.241)	0.169
WBC	Diabetes	Sex	Male: 1.044 (0.986–1.107); Female: 1.110 (1.066–1.155)	**0.046**
WBC	Diabetes	Age	<60: 1.093 (1.045–1.143); ≥60: 1.079 (1.031–1.130)	0.329
WBC	Diabetes	Residence	Rural: 1.083 (1.046–1.122); Urban: 1.105 (1.015–1.203)	0.764
TG	Diabetes	Sex	Male: 1.003 (1.002–1.004); Female: 1.004 (1.003–1.005)	0.491
TG	Diabetes	Age	<60: 1.004 (1.003–1.004); ≥60: 1.003 (1.002–1.004)	0.925
TG	Diabetes	Residence	Rural: 1.003 (1.003–1.004); Urban: 1.004 (1.003–1.005)	0.407
HDL-C	Diabetes	Sex	Male: 0.973 (0.964–0.982); Female: 0.979 (0.972–0.986)	0.201
HDL-C	Diabetes	Age	<60: 0.973 (0.965–0.980); ≥60: 0.981 (0.973–0.989)	0.140
HDL-C	Diabetes	Residence	Rural: 0.977 (0.971–0.983); Urban: 0.976 (0.962–0.990)	0.942
TC	Diabetes	Sex	Male: 1.002 (0.999–1.005); Female: 1.005 (1.002–1.007)	0.145
TC	Diabetes	Age	<60: 1.005 (1.003–1.007); ≥60: 1.002 (0.999–1.005)	0.231
TC	Diabetes	Residence	Rural: 1.004 (1.002–1.006); Urban: 1.004 (1.000–1.008)	0.805
HbA1c	Diabetes	Sex	Male: 1.824 (1.539–2.162); Female: 1.873 (1.698–2.066)	0.900
HbA1c	Diabetes	Age	<60: 1.970 (1.760–2.205); ≥60: 1.831 (1.629–2.058)	0.377
HbA1c	Diabetes	Residence	Rural: 1.918 (1.741–2.113); Urban: 1.768 (1.478–2.114)	0.451
UA	Diabetes	Sex	Male: 1.057 (0.962–1.160); Female: 1.155 (1.060–1.258)	0.176
UA	Diabetes	Age	<60: 1.133 (1.045–1.229); ≥60: 1.078 (0.980–1.186)	0.162
UA	Diabetes	Residence	Rural: 1.081 (1.003–1.166); Urban: 1.187 (1.026–1.374)	0.101
PLT	Hypertension	Sex	Male: 1.001 (1.000–1.002); Female: 1.001 (1.001–1.002)	0.464
PLT	Hypertension	Age	<60: 1.001 (1.001–1.002); ≥60: 1.000 (0.999–1.002)	0.251
PLT	Hypertension	Residence	Rural: 1.001 (1.001–1.002); Urban: 1.000 (0.998–1.003)	0.563
TG	Hypertension	Sex	Male: 1.002 (1.001–1.002); Female: 1.002 (1.001–1.003)	0.984
TG	Hypertension	Age	<60: 1.002 (1.001–1.002); ≥60: 1.002 (1.001–1.003)	0.355
TG	Hypertension	Residence	Rural: 1.002 (1.001–1.002); Urban: 1.003 (1.002–1.004)	**0.011**
HDL-C	Hypertension	Sex	Male: 0.995 (0.989–1.000); Female: 0.992 (0.987–0.997)	0.666
HDL-C	Hypertension	Age	<60: 0.993 (0.988–0.998); ≥60: 0.993 (0.988–0.999)	0.505
HDL-C	Hypertension	Residence	Rural: 0.994 (0.989–0.998); Urban: 0.992 (0.982–1.002)	0.740
TC	Hypertension	Sex	Male: 1.003 (1.001–1.005); Female: 1.001 (0.999–1.002)	0.078
TC	Hypertension	Age	<60: 1.003 (1.001–1.005); ≥60: 1.001 (0.999–1.003)	0.340
TC	Hypertension	Residence	Rural: 1.002 (1.001–1.004); Urban: 1.003 (0.999–1.006)	0.592
HbA1c	Hypertension	Sex	Male: 1.137 (1.054–1.225); Female: 1.135 (1.057–1.218)	0.961
HbA1c	Hypertension	Age	<60: 1.188 (1.117–1.264); ≥60: 1.068 (0.977–1.167)	0.070
HbA1c	Hypertension	Residence	Rural: 1.153 (1.088–1.221); Urban: 1.069 (0.929–1.230)	0.413
UA	Hypertension	Sex	Male: 1.113 (1.046–1.184); Female: 1.093 (1.019–1.172)	0.762
UA	Hypertension	Age	<60: 1.108 (1.042–1.177); ≥60: 1.117 (1.045–1.195)	**0.037**
UA	Hypertension	Residence	Rural: 1.110 (1.054–1.169); Urban: 1.105 (0.982–1.243)	0.936
Creatinine	Hypertension	Sex	Male: 2.136 (1.479–3.085); Female: 1.365 (0.844–2.206)	0.295
Creatinine	Hypertension	Age	<60: 1.885 (1.277–2.781); ≥60: 1.992 (1.223–3.244)	**0.029**
Creatinine	Hypertension	Residence	Rural: 1.866 (1.330–2.618); Urban: 1.370 (0.580–3.239)	0.412

Note. Each row reports stratum-specific HRs and 95% CIs for one subgroup dimension and the corresponding biomarker-by-subgroup interaction p value. Interaction tests were conducted across sex, age (<60 vs. ⩾60 years), and residence (rural vs. urban) for the 32 FDR-significant and event-reliable associations, yielding 96 tests. Bold p values indicate nominal $p < 0.05$. These analyses were exploratory and were not corrected for multiple comparisons. Residence-stratified estimates, particularly for the high-altitude urban subgroup, should be interpreted cautiously because of the small sample size.

Table S8. Complete exploratory mediation results for the 27 candidate pathways selected using prespecified criteria

Disease outcome	Mediator	Cox HR	Cox *p*	Cox *q*	N imputations	ACME	ACME 95% CI	ACME *p*	ADE	ADE 95% CI	ADE *p*
Kidney disease	HbA1c	1.217	<0.001	<0.001	20	0.0000	-0.0005 to 0.0004	0.847	0.0423	0.0182 to 0.0664	<0.001
Dyslipidemia	HbA1c	1.194	<0.001	<0.001	20	0.0004	-0.0009 to 0.0018	0.549	-0.0419	-0.0697 to -0.0141	0.003
Dyslipidemia	UA	1.077	0.004	0.024	20	0.0004	-0.0005 to 0.0014	0.359	-0.0438	-0.0716 to -0.0160	0.002
Dyslipidemia	Creatinine	1.606	0.007	0.040	20	0.0000	-0.0005 to 0.0005	0.919	-0.0438	-0.0715 to -0.0160	0.002
Arthritis	UA	1.086	0.003	0.017	20	0.0002	-0.0012 to 0.0016	0.758	0.0655	0.0234 to 0.1075	0.002
Arthritis	Creatinine	1.763	0.009	0.045	20	0.0000	-0.0007 to 0.0008	0.934	0.0654	0.0230 to 0.1078	0.003
Stroke	PLT	1.002	<0.001	0.002	20	0.0009	-0.0001 to 0.0019	0.068	-0.0024	-0.0227 to 0.0179	0.815
Stroke	TG	1.002	<0.001	<0.001	20	0.0005	-0.0004 to 0.0015	0.258	-0.0069	-0.0263 to 0.0124	0.482
Stroke	HDL-C	0.988	<0.001	0.001	20	-0.0010	-0.0026 to 0.0007	0.250	-0.0061	-0.0254 to 0.0132	0.536
Stroke	HbA1c	1.128	0.002	0.013	20	0.0000	-0.0003 to 0.0004	0.810	-0.0043	-0.0238 to 0.0152	0.664
Stroke	CRP	1.013	<0.001	0.007	20	0.0001	-0.0003 to 0.0005	0.610	-0.0046	-0.0239 to 0.0147	0.640
Heart disease	PLT	1.001	0.003	0.017	20	0.0006	-0.0007 to 0.0019	0.332	-0.0116	-0.0389 to 0.0158	0.407
Heart disease	HbA1c	1.121	<0.001	0.006	20	0.0001	-0.0004 to 0.0006	0.737	-0.0129	-0.0401 to 0.0143	0.353
Lung disease	WBC	1.071	<0.001	0.005	20	0.0015	-0.0011 to 0.0040	0.268	0.0508	0.0218 to 0.0799	<0.001
Diabetes	WBC	1.087	<0.001	<0.001	20	0.0024	0.0004 to 0.0044	0.020	-0.0112	-0.0345 to 0.0121	0.346
Diabetes	TG	1.004	<0.001	<0.001	20	0.0014	0.0002 to 0.0026	0.020	-0.0173	-0.0386 to 0.0040	0.111
Diabetes	HDL-C	0.977	<0.001	<0.001	20	0.0001	-0.0017 to 0.0018	0.947	-0.0163	-0.0378 to 0.0052	0.137
Diabetes	TC	1.004	<0.001	<0.001	20	0.0005	-0.0011 to 0.0020	0.552	-0.0104	-0.0333 to 0.0124	0.371
Diabetes	HbA1c	1.866	<0.001	<0.001	20	0.0016	-0.0014 to 0.0046	0.299	-0.0150	-0.0376 to 0.0076	0.194
Diabetes	UA	1.107	0.002	0.014	20	0.0002	-0.0005 to 0.0009	0.566	-0.0116	-0.0341 to 0.0110	0.315
Hypertension	PLT	1.001	<0.001	0.002	20	0.0007	-0.0009 to 0.0024	0.395	0.0194	-0.0186 to 0.0575	0.317
Hypertension	TG	1.002	<0.001	<0.001	20	0.0019	-0.0003 to 0.0041	0.086	0.0139	-0.0228 to 0.0506	0.457
Hypertension	HDL-C	0.993	<0.001	0.007	20	-0.0003	-0.0038 to 0.0031	0.844	0.0178	-0.0184 to 0.0540	0.335
Hypertension	TC	1.002	0.002	0.013	20	0.0014	-0.0013 to 0.0040	0.306	0.0219	-0.0156 to 0.0594	0.253
Hypertension	HbA1c	1.141	<0.001	<0.001	20	0.0004	-0.0009 to 0.0017	0.537	0.0227	-0.0151 to 0.0604	0.240
Hypertension	UA	1.110	<0.001	<0.001	20	0.0007	-0.0008 to 0.0021	0.374	0.0202	-0.0171 to 0.0574	0.289
Hypertension	Creatinine	1.790	<0.001	0.004	20	0.0001	-0.0009 to 0.0010	0.901	0.0205	-0.0166 to 0.0576	0.279

Note. ACME and ADE were estimated across 20 imputed datasets and are expressed as model-estimated absolute differences in outcome probability for high versus low elevation. The Bonferroni-equivalent threshold was approximately 0.0019; no ACME met this threshold. Proportions mediated are omitted because indirect and direct effects in opposite directions make them non-interpretable. These estimates are exploratory and do not establish causal mediation.

Table S9. Pooled restricted cubic spline nonlinearity tests for continuous elevation and newly reported chronic diseases

Disease outcome	Analytic n	Newly reported cases	High-elevation newly reported cases	Reference elevation, m	Threshold elevation, m	Knots, m	Nonlinearity p value	Nominal p < 0.05
Hypertension	6629	1711	172	0	1500	5, 113, 403, 2192	0.392	No
Heart disease	7925	1124	94	0	1500	5, 107, 403, 2192	0.219	No
Stroke	8816	646	52	0	1500	5, 107, 403, 2192	0.126	No

Disease outcome	Analytic n	Newly reported cases	High-elevation newly reported cases	Reference elevation, m	Threshold elevation, m	Knots, m	Nonlinearity p value	Nominal p < 0.05
Lung disease	8226	967	127	0	1500	5, 107, 403, 2192	0.119	No
Dyslipidemia	7954	1431	100	0	1500	5, 118, 406, 2192	0.768	No
Diabetes	8431	822	58	0	1500	5, 113, 406, 2192	0.837	No
Arthritis	5849	1223	130	0	1500	4, 92, 330, 2192	0.214	No
Kidney disease	8470	590	75	0	1500	5, 107, 392, 2192	0.017	Yes

Note. Models used four knots at outcome-specific elevation percentiles, 20 imputed datasets, MI-IPW, and community-clustered robust covariance estimates. HRs were referenced to 0 m. Among the eight outcomes examined, only kidney disease showed nominal evidence of nonlinearity (p = 0.017). The nonlinearity p values were not adjusted across the eight outcomes.

Table S10. Direct associations of high- versus low-elevation residence with newly reported disease outcomes

Disease outcome	MI-IPW Cox HR (95% CI)	Cox q	Weighted logistic OR (95% CI)	Logistic q
Asthma	1.509 (0.926-2.460)	0.183	1.075 (0.667-1.732)	0.847
Digestive disease	1.456 (1.034-2.051)	0.068	0.949 (0.724-1.245)	0.847
Kidney disease	1.965 (1.460-2.644)	<0.001	1.675 (1.269-2.211)	0.004
Dyslipidemia	0.951 (0.607-1.489)	0.827	0.695 (0.460-1.049)	0.217
Arthritis	1.764 (1.297-2.399)	0.001	1.458 (1.137-1.870)	0.013
Psychiatric problem	1.725 (1.065-2.793)	0.068	1.062 (0.693-1.627)	0.847
Stroke	1.215 (0.830-1.779)	0.410	0.937 (0.681-1.288)	0.847
Heart disease	1.258 (0.913-1.733)	0.261	0.893 (0.657-1.214)	0.767
Lung disease	2.004 (1.493-2.689)	<0.001	1.515 (1.171-1.959)	0.010
Diabetes	1.052 (0.764-1.447)	0.821	0.854 (0.662-1.101)	0.416
Hypertension	1.475 (1.126-1.931)	0.016	1.123 (0.948-1.330)	0.393
Liver disease	1.237 (0.836-1.828)	0.410	0.993 (0.687-1.435)	0.970
Memory problem	0.889 (0.558-1.415)	0.732	0.638 (0.421-0.967)	0.111

Note. Cox estimates used MI-IPW. Weighted logistic models used the primary attrition-weight implementation and provided an alternative analysis that did not rely on exact event timing. q values were corrected across the 13 outcomes within each model family.

Table S11. Summary of sensitivity analyses

Analysis	Key result
MI-IPW Cox sensitivity	180/182 effect directions were concordant with the primary Cox estimates; 34/35 primary FDR-significant findings and 31/32 event-reliable primary FDR-significant findings were retained. The creatinine–dyslipidemia association remained positive but moved slightly above the FDR threshold (q = 0.061); no other primary FDR classification changed.
Complete-weight-case Cox sensitivity	168/182 directions concordant; 28/35 FDR findings retained.
Weighted logistic sensitivity	167/182 directions concordant; 26/35 primary FDR findings and 24/32 event-reliable FDR findings retained.
Log/SD biomarker sensitivity	178/182 directions concordant; 32/35 FDR findings retained.
TG log-transformation sensitivity	All 13 TG–disease associations retained the same direction, and all five primary FDR-significant TG associations remained FDR-significant after natural-log transformation and standardization.
Proportional hazards tests	13 exposure-specific and 23 global tests had nominal p < 0.05; none remained significant after FDR correction.
HbA1c-diabetes exclusion	After excluding baseline HbA1c ≥6.5%, Cox HR=3.549 (95% CI 2.853-4.417) and logistic OR=3.805 (95% CI 3.009-4.812).
Alternative elevation thresholds	Kidney disease remained FDR-significant at 1500, 2000, and 2500 m; lung disease remained significant at 1500 and 2000 m.

Note. This table summarizes the sensitivity and model-diagnostic analyses. The classification of analyses as prespecified, exploratory, or post hoc is described in the main-text Methods.

Table S12. Nominal ACME findings from the temporal-order sensitivity analysis after excluding participants who reported the corresponding disease by Wave 3

Disease	Mediator	Analytic n	Post-Wave-3 events	ACME (95% CI)	ACME p	BH q	ADE (95% CI)	ADE p
Diabetes	WBC	7347	477	0.00146 (0.00003 to 0.00290)	0.046	0.616	-0.00052 (-0.01927 to 0.01822)	0.956
Diabetes	TG	7350	474	0.00098 (0.00011 to 0.00184)	0.026	0.616	-0.00425 (-0.02183 to 0.01334)	0.636

Note. All 27 candidate pathways were re-evaluated after excluding participants who reported the relevant disease by Wave 3. Only the two pathways with nominal ACME $p < 0.05$ are shown. Neither pathway, and none of the 27 pathways overall, remained significant after BH-FDR or Bonferroni correction. These analyses are exploratory and do not establish causal mediation.

Table S13. Missingness of biomarkers and model covariates

Variable	Type	Baseline missing, n (%)	Longitudinal missing, n (%)
Hb	Biomarker	301 (2.6%)	213 (2.3%)
HCT	Biomarker	8 (0.1%)	8 (0.1%)
WBC	Biomarker	304 (2.7%)	214 (2.3%)

MCV	Biomarker	310 (2.7%)	222 (2.4%)
PLT	Biomarker	301 (2.6%)	212 (2.3%)
TG	Biomarker	187 (1.6%)	146 (1.6%)
HDL-C	Biomarker	179 (1.6%)	139 (1.5%)
LDL-C	Biomarker	198 (1.7%)	158 (1.7%)
TC	Biomarker	186 (1.6%)	144 (1.6%)
HbA1c	Biomarker	135 (1.2%)	89 (1.0%)
UA	Biomarker	178 (1.6%)	139 (1.5%)
CRP	Biomarker	178 (1.6%)	139 (1.5%)
BUN	Biomarker	180 (1.6%)	139 (1.5%)
Creatinine	Biomarker	204 (1.8%)	158 (1.7%)
Age	Covariate	0 (0.0%)	0 (0.0%)
Sex	Covariate	0 (0.0%)	0 (0.0%)
Marital status	Covariate	2 (0.0%)	2 (0.0%)
Education	Covariate	0 (0.0%)	0 (0.0%)
Rural residence	Covariate	12 (0.1%)	9 (0.1%)
BMI	Covariate	1805 (15.9%)	1347 (14.5%)
Smoking	Covariate	267 (2.3%)	187 (2.0%)
Alcohol use	Covariate	57 (0.5%)	42 (0.5%)
Annual individual earnings	Covariate	73 (0.6%)	51 (0.6%)

Note. Baseline n =11,371 and longitudinal n =9270. Baseline biomarkers used as study exposures or outcomes were not imputed.

Table S14. FDR-significant direct disease associations at the primary and alternative elevation thresholds using the primary attrition-weight implementation

Threshold, m	Model	Disease outcome	Effect (95% CI)	q
1500	Cox HR	Kidney disease	1.975 (1.460-2.671)	<0.001
1500	Logistic OR	Kidney disease	1.675 (1.269-2.211)	0.004
1500	Cox HR	Arthritis	1.732 (1.266-2.370)	0.003
1500	Logistic OR	Arthritis	1.458 (1.137-1.870)	0.013
1500	Cox HR	Lung disease	1.852 (1.391-2.465)	<0.001
1500	Logistic OR	Lung disease	1.515 (1.171-1.959)	0.010
2000	Cox HR	Kidney disease	1.967 (1.327-2.916)	0.005
2000	Logistic OR	Kidney disease	1.776 (1.256-2.513)	0.015
2000	Cox HR	Lung disease	1.922 (1.360-2.715)	0.003
2000	Logistic OR	Lung disease	1.654 (1.194-2.292)	0.016
2500	Cox HR	Kidney disease	2.463 (1.532-3.960)	0.003
2500	Logistic OR	Kidney disease	2.086 (1.410-3.085)	0.003
2500	Logistic OR	Diabetes	0.570 (0.387-0.839)	0.029

Note. Only associations with $q < 0.05$ are shown. Cox and logistic models used the prespecified primary attrition-weight implementation at each elevation cut point. The MI-IPW estimates for the primary 1500 m exposure definition are reported in Table S10. Threshold-specific biomarker results are available from the corresponding author upon reasonable request.

Table S15. Diagnostics for attrition weights

Weight implementation	n	Mean	SD	Minimum	Median	Maximum	ESS
Original weights before truncation	7238	0.976	0.137	0.835	0.934	2.707	7098
Original weights after P1-P99 truncation	7238	0.973	0.120	0.854	0.934	1.511	7130
Primary implementation after unavailable weights were assigned a unit weight	9270	0.979	0.107	0.854	0.968	1.511	9162
MI-IPW after P1-P99 truncation, mean across 20 imputations	9270	0.995	0.143	0.860	0.948	1.665	9082

Note. Original-weight rows summarize the complete-case participation model before and after 1st–99th percentile truncation. In the primary implementation, 2032 Wave 4 participants without calculable complete-case participation weights were assigned a unit weight to retain them in the analytic cohort. A unit weight indicated that no additional model-based attrition correction was applied and did not represent an estimated participation probability of 1. MI-IPW values are

means across the 20 imputed datasets and were available for all 9270 Wave 4 participants. ESS, effective sample size; MI-IPW, multiple-imputation inverse-probability weighting; P1–P99, 1st–99th percentile.

Supplementary Figures

Figure S1. Hemoglobin and hematocrit patterns in the baseline cross-sectional analytic sample

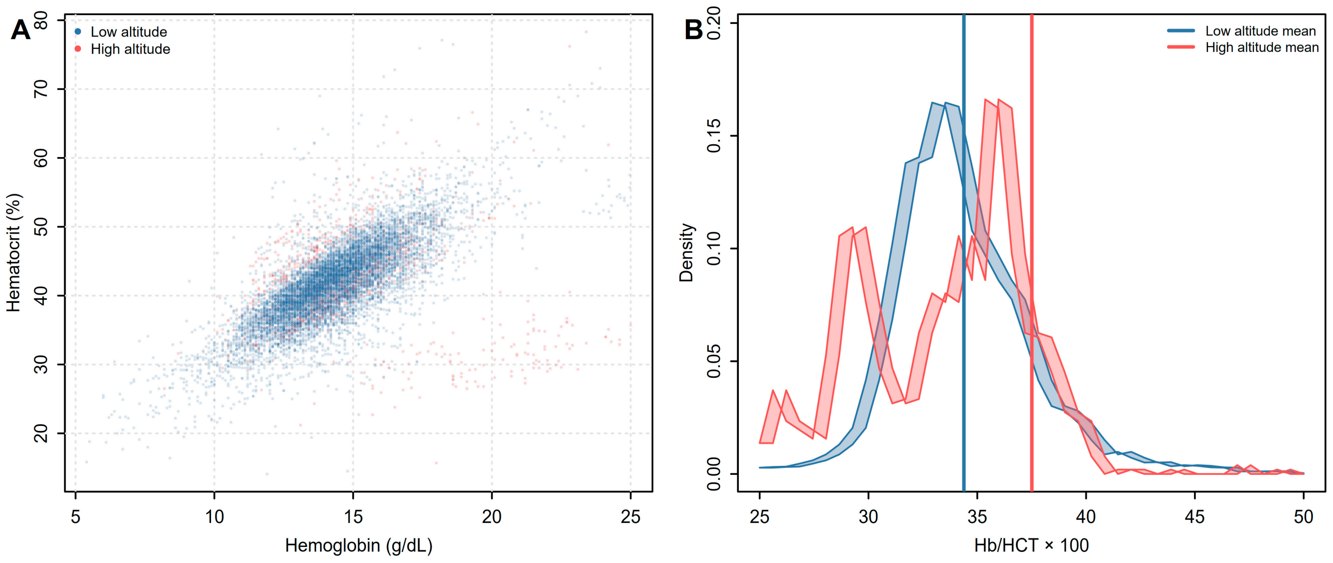

Note. Panel A shows the joint distribution of hemoglobin and hematocrit in the baseline cross-sectional analytic sample. Panel B shows the distribution of the Hb-to-HCT ratio in the same sample, providing descriptive context for the higher hemoglobin level and comparatively small hematocrit difference observed between elevation groups. Panel B is displayed over the 0.5th – 99.5th percentile range of the Hb-to-HCT ratio.

Figure S2. Assumed directed acyclic graph for the exploratory mediation analysis.

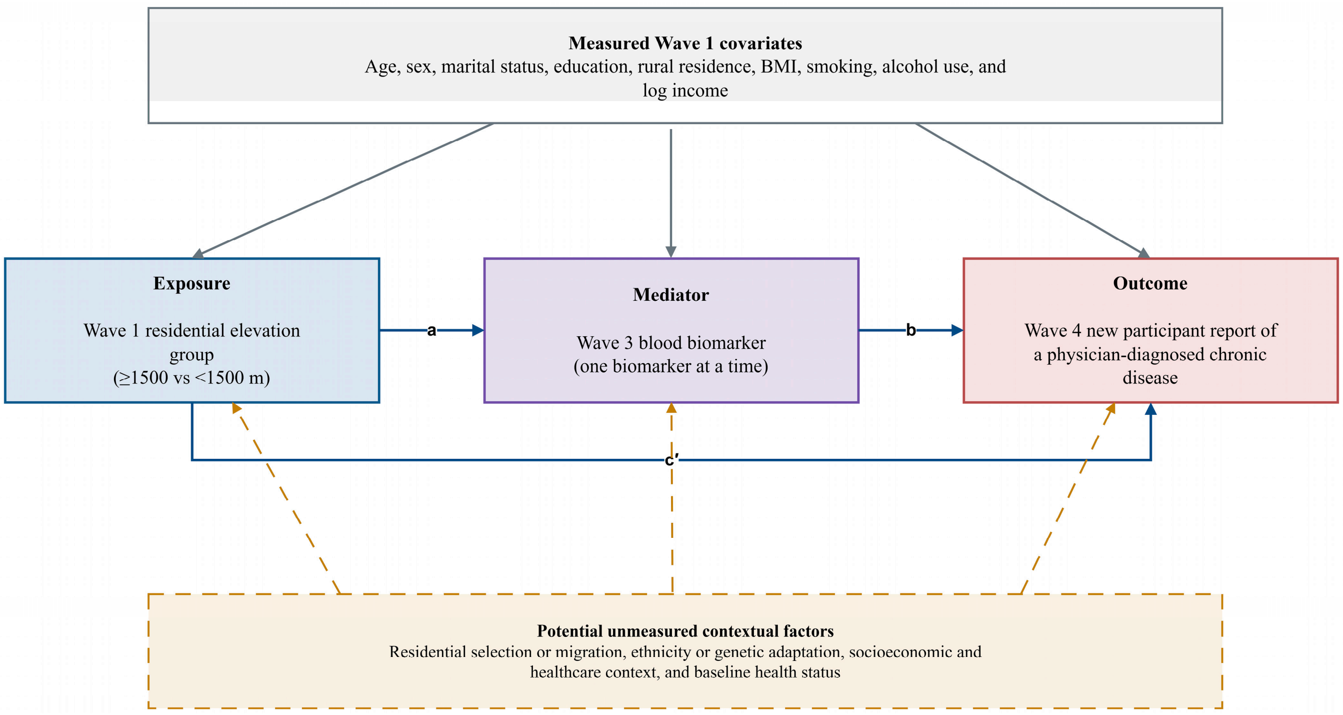

Note. Wave 1 residential elevation group was treated as the exposure, each Wave 3 blood biomarker was evaluated separately as a potential mediator, and a new Wave 4 participant report of a physician-diagnosed chronic disease was

treated as the outcome. Paths a, b, and c′ denote the exposure–mediator, mediator–outcome, and direct exposure–outcome relations, respectively. Models adjusted for measured Wave 1 covariates and used inverse-probability weighting for Wave 4 attrition. Dashed arrows indicate potential residual confounding by contextual factors not fully captured in CHARLS. The diagram represents the assumed analytical structure and does not establish causal mediation; unmeasured confounding, exposure misclassification, and reverse causation remain possible.
